# Autoregulation of the *Drosophila* Noncoding *roX1* RNA Gene

**DOI:** 10.1371/journal.pgen.1002564

**Published:** 2012-03-15

**Authors:** Chiat Koo Lim, Richard L. Kelley

**Affiliations:** 1 Program in Developmental Biology, Baylor College of Medicine, Houston, Texas, United States of America; 2 Department of Molecular and Human Genetics, Baylor College of Medicine, Houston, Texas, United States of America; Massachusetts General Hospital, Howard Hughes Medical Institute, United States of America

## Abstract

Most genes along the male single X chromosome in *Drosophila* are hypertranscribed about two-fold relative to each of the two female X chromosomes. This is accomplished by the MSL (male-specific lethal) complex that acetylates histone H4 at lysine 16. The MSL complex contains two large noncoding RNAs, *roX1* (*RNA on X*) and *roX2*, that help target chromatin modifying enzymes to the X. The *roX* RNAs are functionally redundant but differ in size, sequence, and transcriptional control. We wanted to find out how *roX1* production is regulated. Ectopic DC can be induced in wild-type (*roX1^+^ roX2^+^*) females if we provide a heterologous source of MSL2. However, in the absence of *roX2*, we found that *roX1* expression failed to come on reliably. Using an *in situ* hybridization probe that is specific only to endogenous *roX1*, we found that expression was restored if we introduced either *roX2* or a truncated but functional version of *roX1*. This shows that pre-existing *roX* RNA is required to positively autoregulate *roX1* expression. We also observed massive *cis* spreading of the MSL complex from the site of *roX1* transcription at its endogenous location on the X chromosome. We propose that retention of newly assembled MSL complex around the *roX* gene is needed to drive sustained transcription and that spreading into flanking chromatin contributes to the X chromosome targeting specificity. Finally, we found that the gene encoding the key male-limited protein subunit, *msl2*, is transcribed predominantly during DNA replication. This suggests that new MSL complex is made as the chromatin template doubles. We offer a model describing how the production of *roX1* and *msl2*, two key components of the MSL complex, are coordinated to meet the dosage compensation demands of the male cell.

## Author Summary

In fruit flies, the male undergoes dosage compensation to increase the transcriptional output of its single X chromosome to match that of the females' two copies. A large ribonucleoprotein complex that consists of two large noncoding RNA, *roX1* and *roX2*, and at least five MSL (male specific lethal) chromatin-modifying proteins is responsible. We were surprised to find that transcription of new *roX1* RNA failed in the absence of pre-existing *roX* RNA. When we introduced either *roX2* or the truncated but functional version of *roX1*, expression of endogenous wild-type *roX1* gene was restored. We propose that this mechanism is essential for ensuring sustained dosage compensation in males and perhaps also for bursts of new MSL complex at each cell cycle. Thousands of large noncoding RNAs have recently been discovered in vertebrates, many of which are associated with chromatin remodeling enzymes. We postulate that some of these will face similar regulatory and functional demands as the *roX* RNAs and may have evolved comparable strategies to control their production.

## Introduction

Some long noncoding RNAs have the ability to recruit chromatin modifying enzymes to specific genes thereby controlling their expression [1]. Other noncoding RNAs behave as transcriptional enhancers to flanking protein coding genes [2]. The two *roX* RNAs that participate in dosage compensation of the single male X chromosome in *Drosophila* are some of the best characterized examples of noncoding RNAs that target chromatin remodeling enzymes to large domains [3]. The *roX* RNAs assemble into a complex containing at least five MSL protein subunits that bind actively transcribed genes along the male X chromosome, but not autosomes or the two X chromosomes in females [4]. This has been termed the dosage compensation complex or the MSL complex. One function of the complex is acetylation of histone H4 at lysine 16, carried out by the MOF (males absent on first) histone acetyltransferase resulting in an essential ∼two-fold increase in transcription [5]–[8]. Another modification is ubiquitylation of histone H2B at K34 by the MSL2 RING finger protein [9].

Flies carry two *roX* genes that differ greatly in size and sequence [10]. The *roX1* gene is located on the X chromosome at polytene band 3F and produces a 3.7 kb RNA. The *roX2* gene is located at 10C on the X and makes a ∼600 nt RNA. Both RNAs ‘paint’ the length of the male X in a banded pattern [11]. The only obvious sequence similarity between them is limited to short repeated elements near the 3′ end of each gene [12]. These repeats are essential for function and predicted to fold into conserved secondary structures [13], [14]. Neither *roX* RNA is maternally deposited in eggs. Zygotic transcription of *roX1* RNA occurs in both male and female embryos beginning at blastoderm [15]. Females lose *roX1* RNA midway through embryogenesis, but males maintain expression through adulthood. By contrast, *roX2* RNA first appears a few hours after *roX1* but only in male embryos [16]. Despite the vast differences in size, sequence, and regulation, the two *roX* RNAs are functionally redundant [17].

Little is known about how production of the *roX* RNAs and MSL protein subunits are coordinated. Unusual *cis* spreading behavior of MSL complex from sites of autosomal *roX* transgene has been attributed to cotranscriptional assembly of free MSL subunits onto growing nascent *roX* transcripts [13], [18], [19], although direct biochemical evidence is lacking. Most MSL protein subunits are made in both males and females, except for MSL2 which is translationally repressed in females by the action of SXL [20], [21]. MSL2 is a RING finger protein that binds DNA in a sequence independent manner through a second cysteine-rich motif [22]. The *H83M2* transgene removes the 5′ and 3′ UTRs containing SXL binding sites from *msl2* mRNA and has been used extensively to drive ectopic MSL2 expression in females [5], [11], [15], [17], [23]–[29]. Forcing females to make MSL2 using the *H83M2* transgene induces production of *roX* RNAs, resulting in ectopic dosage compensation that is toxic to females [15], [30]. These observations led to the idea that MSL2 protein alone, or acting with the other MSL proteins drives transcription of *roX1* RNA [31]–[33].

We reexamined the question of how MSL proteins regulate transcription of the *roX1* gene using flies missing the *roX2* locus. Deleting *roX2* allowed us to study expression of the wild type endogenous *roX1* gene without the confounding effects of a second functionally redundant RNA species. In this way we found an RNA-dependent autoregulatory loop controlling *roX1* expression. We propose that the early burst of *roX1* transcription at blastoderm initiates this cycle. Furthermore, production of the key male-limited MSL2 protein subunit is not only regulated at the translational level as has been extensively documented, but we find *msl2* transcription is associated with DNA replication. We propose a model where pre-existing *roX* RNA, assembled in mature MSL complexes, drives bursts of *roX1* transcription during S phase when its chromatin target is doubling.

## Results

### *roX1* transcription is dependent on *roX2* RNA

**Figure 1:**
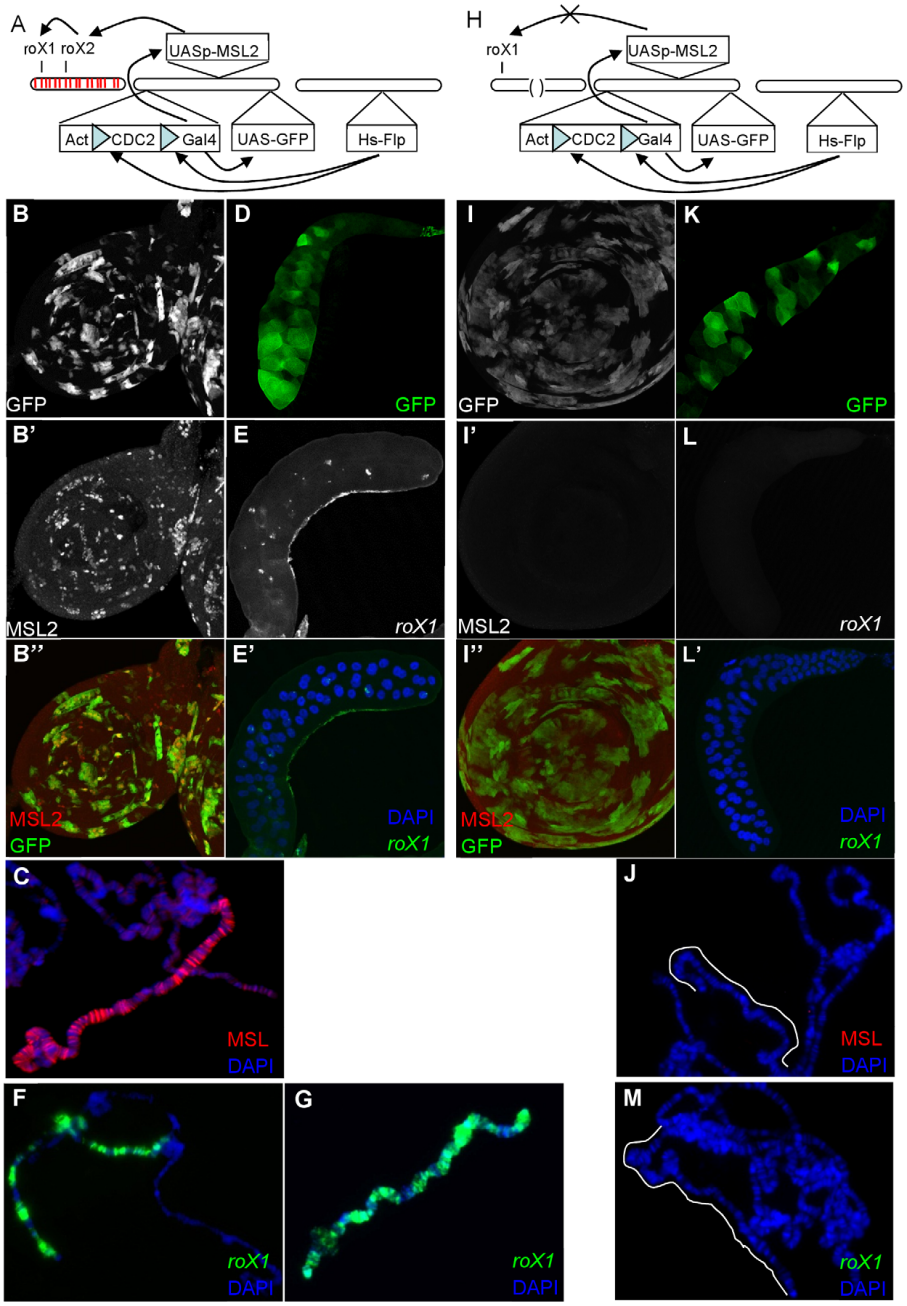
MSL proteins alone cannot drive *roX1* expression late in development. A) 4 day old larvae were heatshocked to induce expression of Flp, resulting in the removal of the blocking sequence from GAL4 and subsequent expression of both MSL2 and GFP. MSL2 is expected to initiate *roX* transcription and MSL complex assembly. (B) GFP+ clones mark imaginal disc cells that have successfully removed the blocking sequences from *GAL4* (B′–B″). Induction of MSL2 results in punctate subnuclear foci in imaginal disc cells. (C) MSL2 immunostaining of polytene chromosome shows late MSL2 paints the entire X chromosome. (D) Whole salivary gland showing GFP induced in some cells. (E–E′) *roX1* FISH of whole mount of similar GFP+ salivary glands or (F) polytene squashes shows successful induction of *roX1* expression in a subset of cells. (G) *roX1* FISH of wildtype males (H) The same experiment was repeated in *roX1^+^roX2^−^* larvae. However, in the absence of *roX2*, MSL2 fails to drive *roX1* expression. (I) Despite the presence of GFP+ (late MSL2 expressing) cells, MSL2 is not detectable over the X in (I′–I″) imaginal disc cells or (J) polytene chromosomes. (K) Whole salivary gland showing successful GFP expression in *roX1^+^roX2^−^* larvae. (L–L′) Expression of *roX1* is never observed painting the X or as nascent transcripts at band 3F in separately processed GFP+ glands or on (M) polytene squashes.

Male embryos normally establish dosage compensation by the onset of gastrulation [16], [34]. Ectopic expression of MSL2 in females leads to *roX1* transcription and dosage compensation [15]. We asked whether dosage compensation can only be initiated during early embryogenesis, or could it be artificially started later during larval development. To achieve that, we used an inducible Flp-out system [35] to create clones of GAL4 expressing cells on day 4 AEL (after egg laying) that in turn drove expression of *UAS-GFP* and *UAS-MSL2* in female larvae (Figure 1A and Methods). The purpose of this was to assay females in which the early burst of *roX1* RNA had decayed away leaving cells devoid of any *roX* RNA. When no clones were induced, we never observed GFP or MSL painting in any cell showing that expression was tightly blocked prior to induction (data not shown). Third instar larvae in which late GAL4+ clones were induced displayed overlapping expression of GFP and MSL2 (Figure 1B–1B″). More importantly, MSL2 appeared as subnuclear punctate staining in imaginal disc cells suggesting that it was concentrated on the X chromosome (Figure 1B″). MSL2 immunostaining of polytene squashes confirmed binding along the X (Figure 1C). Unfortunately, we were unable to perform GFP immunostaining and reliable *roX1* FISH (Fluorescence *in situ* hybridization) in the same glands as the proteinase K treatment necessary to expose *roX* RNA often destroyed protein epitopes. We took comparable salivary glands from GFP positive larvae (Figure 1D) and processed them for *roX1* FISH. The results show that *roX1* transcription was successfully induced (Figure 1E–1F), consistent with previous reports that MSL2 drives *roX1* transcription [15], and confirms an earlier report that dosage compensation can be initiated long after it normally occurs [13]. Figure 1G shows a wild type male X for comparison.

We repeated the experiment in *roX1* ^+^ *roX2* ^−^ females (Figure 1H). While GFP+ clones were recovered at similar frequencies indicating successful MSL2 induction (Figure 1I, 1K), no MSL2 accumulation was observed in imaginal discs (Figure 1I′) or on polytene chromosomes (Figure 1J). We will later demonstrate that the failure to detect MSL2 is due to reduced protein stability in the absence of *roX* RNA. More importantly, we also could not detect *roX1* expression in any cell. Absence of *roX1* RNA might be attributed to poor RNA stability or transcription failure. We favor the latter since even minute amounts of *roX1* transcription can be readily detected when MSL complex accumulates over the *roX1* gene [13]. Moreover, we could not detect nascent transcripts from the *roX1* locus in these animals although such nascent *roX1* transcripts were easily detected in other genotypes (Figure 1L–1L′, 1M). This argues that although late MSL2 readily switches on *roX2*, it is not sufficient alone or with the other MSL subunits to drive expression of the *roX1* gene when *roX2* is absent. Without any *roX* RNA, cells rapidly destroy the ectopic MSL2 as well.

**Figure 2:**
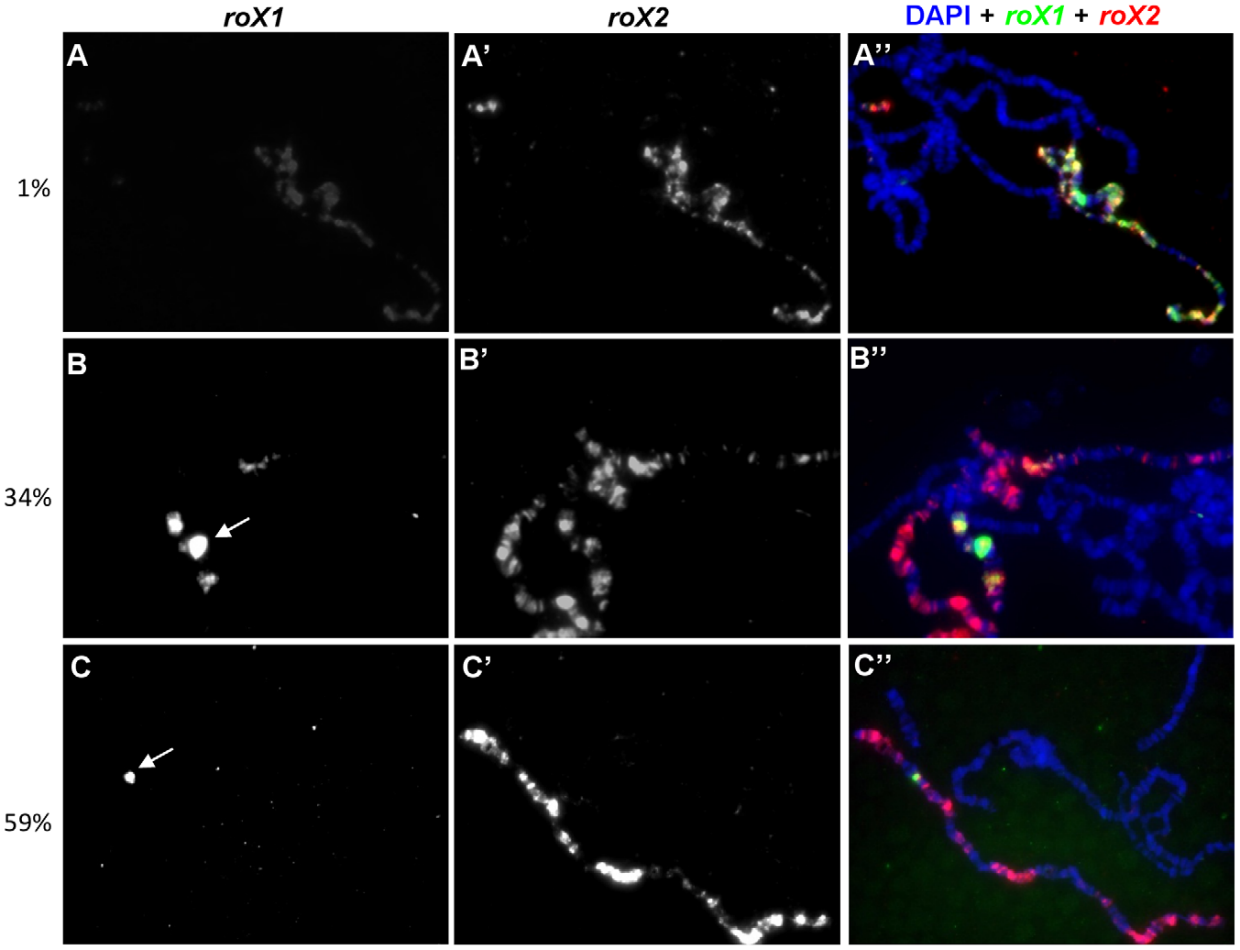
Late induction of *roX1* expression requires *roX2* RNA. In nuclei where dosage compensation was successfully turned on after late *msl2* induction, extensive *roX2* was observed painting the entire X chromosome. (A) However, only 1% of the chromosomes showed extensive *roX1* painting. 34% and 59% of chromosomes showed *roX1* expression confined to several Mbp around (B) or just at the endogenous *roX1* locus (C), respectively. The remaining chromosomes (6%) had no *roX1* expression despite the presence of *roX2* (data not shown). *roX1* and *roX2* were detected by biotin (green, A–C) and digoxigenin (red, A′–C′) labeled antisense riboprobes, respectively. The merged figure is shown in A″–C″. White arrows denote the endogenous *roX1* locus at band 3F.

To further test our hypothesis that late expression of the endogenous *roX1* locus depends on *roX2* RNA, we returned to wildtype (*roX1* ^+^ *roX2^+^*) females to perform RNA *in situ* hybridization for both RNAs in the same nuclei. Late induction of MSL2 results in *roX2* RNA painting the length of X chromosomes in many cells (Figure 2A′). By contrast, *roX1* painting over the full X chromosome was seen in only a small minority of nuclei (Figure 2A). A much more common pattern was *roX2* over the entire X while *roX1* expression confined to either several Mbp (Figure 2B–2B′) around or just at the endogenous *roX1* locus at polytene band 3F (Figure 2C–2C′). This suggests that *roX2* expression reliably follows MSL2 induction, but *roX1* expression lags. Delayed *roX1* expression might be explained if fully functional MSL complex containing *roX2* RNA must first assemble before transcription of *roX1* can occur. We conclude that *roX2* RNA, presumably packaged in mature MSL complexes, is necessary to initiate transcription from the endogenous *roX1* gene late in development.

### Autoregulation of *roX1* expression

Finding unusually late activation of the *roX1* gene required preexisting *roX2* RNA, we wondered if *roX1* RNA could also perform the same role leading to a positive autoregulatory loop. To answer this question, we used a fly stock displaying an unusual mosaic pattern of dosage compensation.

**Figure 3:**
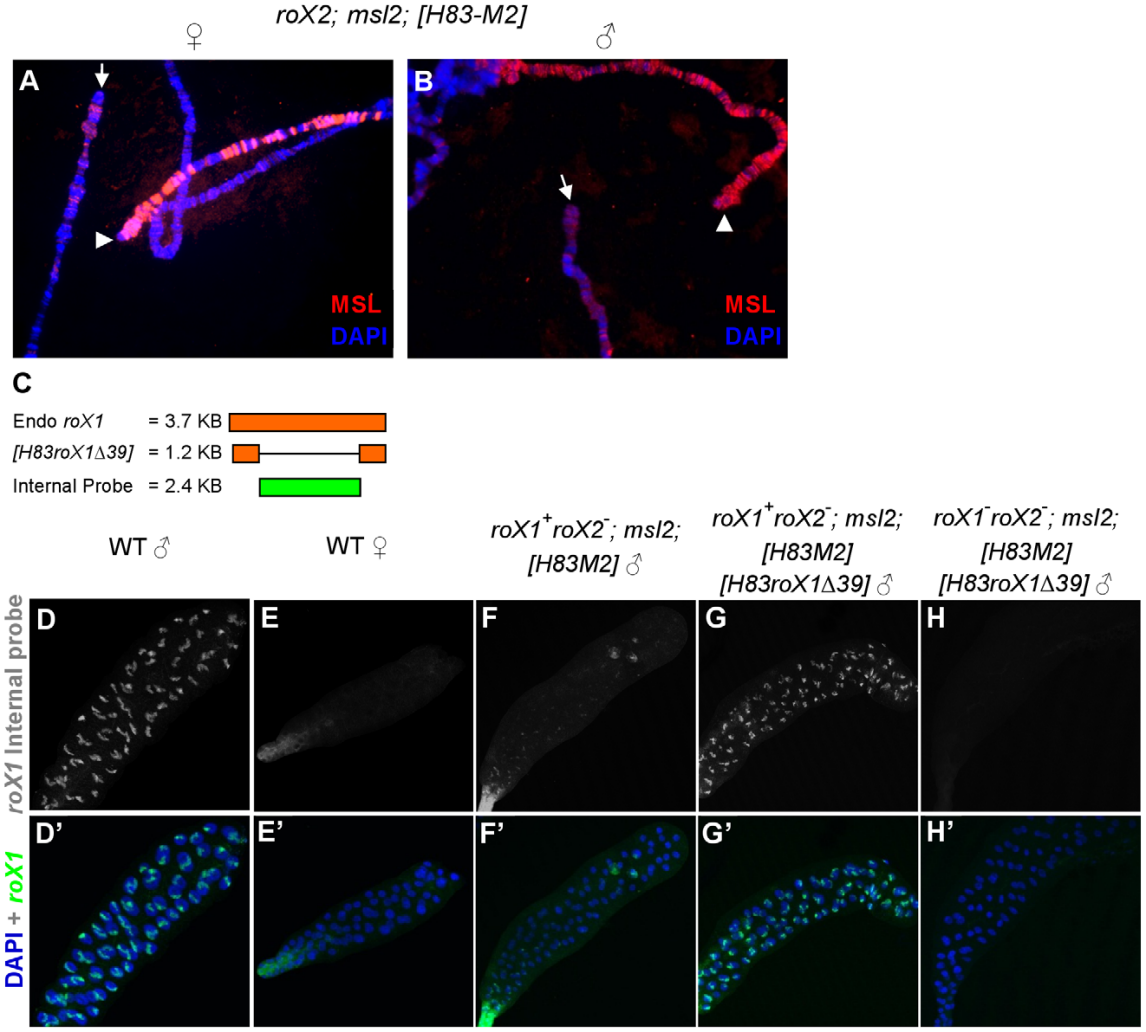
*roX1* RNA is needed to sustain endogenous *roX1* transcription in males. X chromosomes from neighboring cells display a mosaic pattern in which the MSL complex either succeeded (arrowhead) or failed (arrow) to paint the X from *roX2*; *msl2*; *H83M2* female (A) and male (B) salivary glands. (C) Endogenous *roX1* and *H83roX1Δ39* transcripts (Orange) and antisense riboprobe recognizing only full length *roX1* (green). Whole mount *roX1* FISH using the internal probe on salivary glands from (D) wild type male, (E) wild type female, (F) *roX1^+^ roX2^−^*/Y; *msl2*; *H83M2* mosaic male, (G) *roX1^+^roX2^−^*/Y; *msl2; H83M2 H83-roX1Δ39*/+ male, (H) *roX1^−^ roX2 ^−^*/Y; *msl2; H83M2 H83-roX1Δ39*/+ male. The X chromosomes in G are fully painted in all cells with MSL complex relying upon *roX1-Δ39* RNA (Figure S1D), but the truncated *roX1* RNA is not recognized by the internal probe.

The *H83M2* transgene makes MSL2 constitutively using the *hsp83* promoter [30]. It lacks the regulatory 5′ and 3′ *msl2* UTRs and drives ectopic dosage compensation in 100% of female cells. Females carrying a *roX1* deletion also showed MSL X chromosome painting utilizing *roX2* RNA in all nuclei (data not shown). However, an entirely different result was obtained in *H83M2* females missing only *roX2*. Roughly half the nuclei adopted a fully male-like pattern of dosage compensation utilizing *roX1* RNA, while the other half lacked dosage compensation (Figure 3A and personal communication Art Alekseyenko). The X chromosomes of these negative cells showed very weak staining for MSL2, similar to the situation found in *roX1 roX2* double mutant animals [17]. Males also exhibited a similar mosaic phenotype when their only source of MSL2 was the *H83M2* transgene demonstrating that failed dosage compensation cannot be due to SXL or some other female factor (Figure 3B). It is unclear how two adjacent cells that are genetically identical containing a full set of MSL subunits adopt opposite dosage compensation fates. However, this fortuitous observation allowed us to test whether failure to activate the endogenous *roX1* gene might explain lack of dosage compensation in some cells.

In order to test whether pre-existing *roX1* RNA is necessary to drive continued transcription of the *roX1* gene, we used a 1.2 kb *roX1* minigene, *H83roX1-Δ39*, that is able to form partially active MSL complexes (Figure 3C) [13]. We designed a probe that recognizes only the internal sequence of the endogenous *roX1* RNA missing from *H83roX1-Δ39*. In this way, we could selectively visualize the expression of only the endogenous *roX1* RNA in animals also making the shorter transgenic *H83roX1-Δ39* RNA (Figure 3D, 3H and Figure S1C). The *roX2*; *H83M2* males (*roX1* ^+^ *roX2^−^/Y*; *msl2^−^*; *H83M2/+*) displayed the same mosaic *roX1* pattern (Figure 3F) typically seen in polytene squashes (Figure 3A and 3B). When we introduced *H83roX1-Δ39* into these *roX2 H83M2* males, the expression of full-length *roX1* RNA made from the endogenous locus was restored to all cells (Figure 3G and Figure S1B, lane 3). In addition, while the distribution of *roX1* RNA was often limited to a single band or several Mbp around 3F in nuclei from the mosaic *H83M2* males (Figure S1E–S1F), the endogenous, full-length *roX1* RNA coated the entire length of the X chromosome in nearly all cells when the constitutively expressed *H83roX1-Δ39* transgene was also present, (Figure S1G). The *H83roX1-Δ39* transgene was ineffective in *H83M2* females (Figure S1B, lane 4), perhaps because females have two X chromosomes, depressed MSL1 expression [36] and so require more stimulatory activity than *H83roX1-Δ39* can supply.

We conclude that the unexpected mosaic pattern found in *roX2 H83M2* animals results from a failure of the wild type *roX1* gene to respond to MSL proteins. Providing a constitutive source of *roX1* RNA is sufficient to reliably drive transcription of the endogenous wild type *roX1* gene. Taken together, these results support an autoregulatory model where new transcription of the wild type *roX1* gene requires pre-existing RNA, either *roX1* or *roX2*, in addition to MSL proteins. We postulate that the early MSL-independent burst of *roX1* RNA made at blastoderm normally assembles the first MSL complexes needed to set up the future maintenance of *roX1* transcription in adult males.

### The mosaic expression of *roX1* is not limited to the polytene chromosomes

The mosaic pattern of dosage compensation described above has been reported before. Certain hypomorphic alleles of *Sxl* unreliably initiate sex determination and so contain a mixture of XXAA cells that either correctly adopt a female fate repressing dosage compensation, or mistakenly choose a male fate and paint their X chromosomes with MSL complex [37]. The mechanism underlying the mosaic pattern of dosage compensation found in *roX2 H83M2* animals studied here must be different, and we set out to understand its basis.

**Figure 4:**
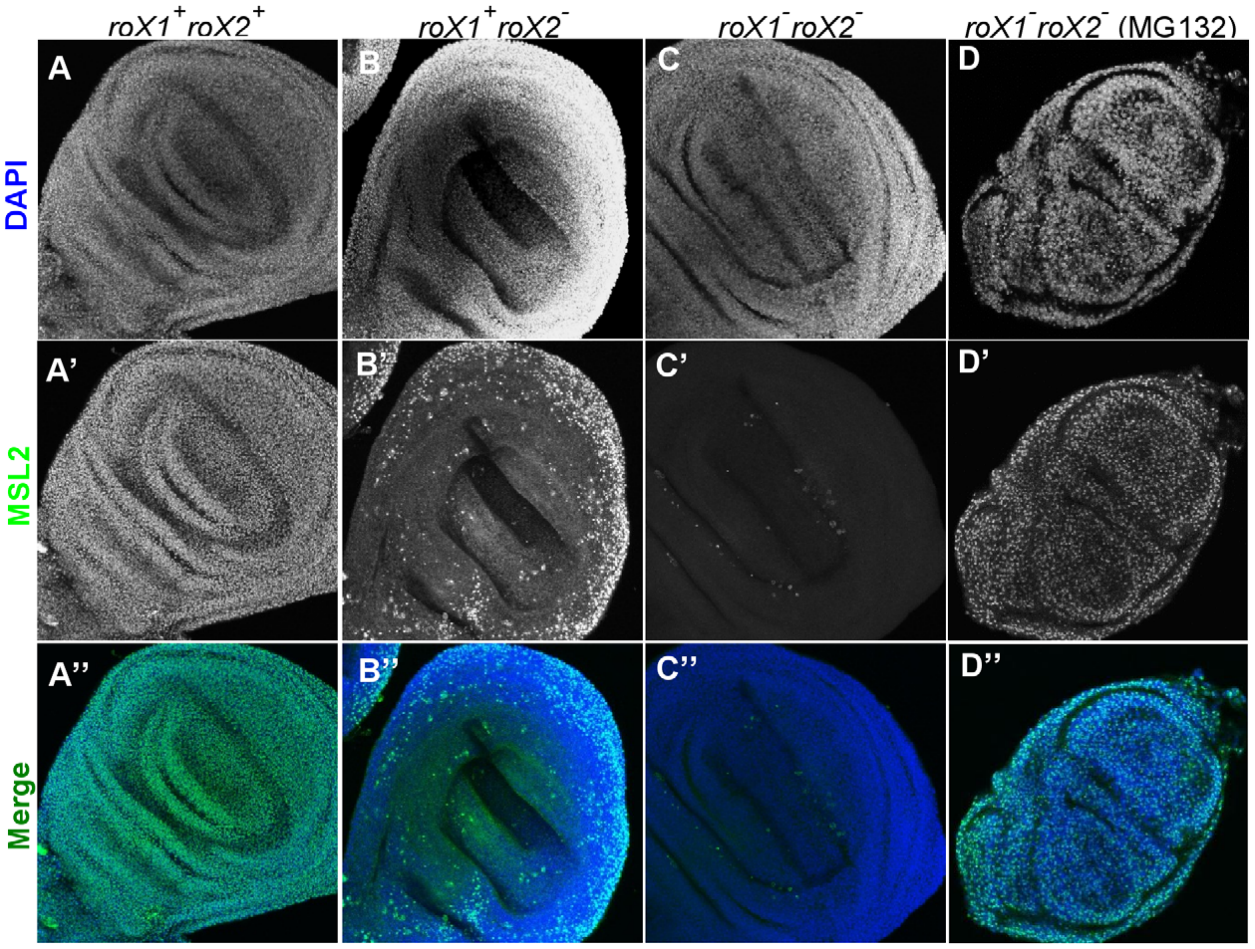
Dosage compensation fails in many diploid cells relying exclusively on *roX1* and *H83M2*. (A–A″) Males and females relying entirely on *H83M2* showed subnuclear MSL2 staining in all wing imaginal disc cells when both *roX1* and *roX2* RNAs were present. (B–B″) Many cells lacked dosage compensation if *roX1* ^+^ was the only source of *roX* RNA. (C–C″) MSL2 did not accumulate over the X when both *roX1* and *roX2* were absent. (D–D″) Nuclear staining of MSL2 was easily detected in the absence of *roX* RNA after treatment with MG132, a proteasome inhibitor.

We first tested if the mosaic pattern was a peculiarity of polytene cells or a general feature in most tissues of these animals. When we performed MSL2 immunostaining on diploid imaginal discs, MSL2 protein decorated a subnuclear domain presumed to be the X chromosome in all the imaginal disc cells when both *roX* RNAs were present in female cells carrying the *H83M2* transgene (Figure 4A). Again, *roX1^−^ roX2^+^* animals also had no trouble establishing dosage compensation in all the cells (data not shown). However, when *roX2* was deleted, only a fraction of the cells displayed dosage compensation utilizing *roX1* RNA (Figure 4B–4B″), similar to the spotty pattern observed in salivary glands (Figure 3A, 3B and 3F). When both *roX* genes were deleted, no MSL2 staining was detected (Figure 4C–4C″). We had expected that removing *roX* RNA would produce a diffuse nuclear cloud of MSL2 protein unable to bind to the X chromosome. Either diffuse MSL2 protein stains too weakly to detect with our antibodies, or MSL2 protein is unstable without *roX* RNA. The latter is likely to be the case since MSL2 was previously shown to be unstable when not packaged into MSL complexes [29]. To directly test this interpretation, we incubated the imaginal discs and salivary glands with MG132, a proteasome inhibitor, before performing MSL2 immunostaining. Strong nuclear MSL2 staining reappeared (Figure 4D–4D″) in the imaginal disc, showing that MSL2 is synthesized in the absence of *roX* RNA, but fails to accumulate due to rapid turnover. MSL2 protein stabilized by MG132 showed a dramatically different staining pattern covering all polytene chromosomes, rather than being restricted to the X (Figure S2). This shows that free MSL2 subunits have a general affinity for all chromatin in agreement with earlier biochemical work [22]. Apparently flies have evolved a mechanism to efficiently destroy any MSL2 subunits that fail to assemble into complexes with *roX* RNA. A somewhat similar situation was reported for the MSL1 subunit. Massively over expressed MSL1 transiently paints all chromosome arms but is quickly lost [36].

**Figure 5:**
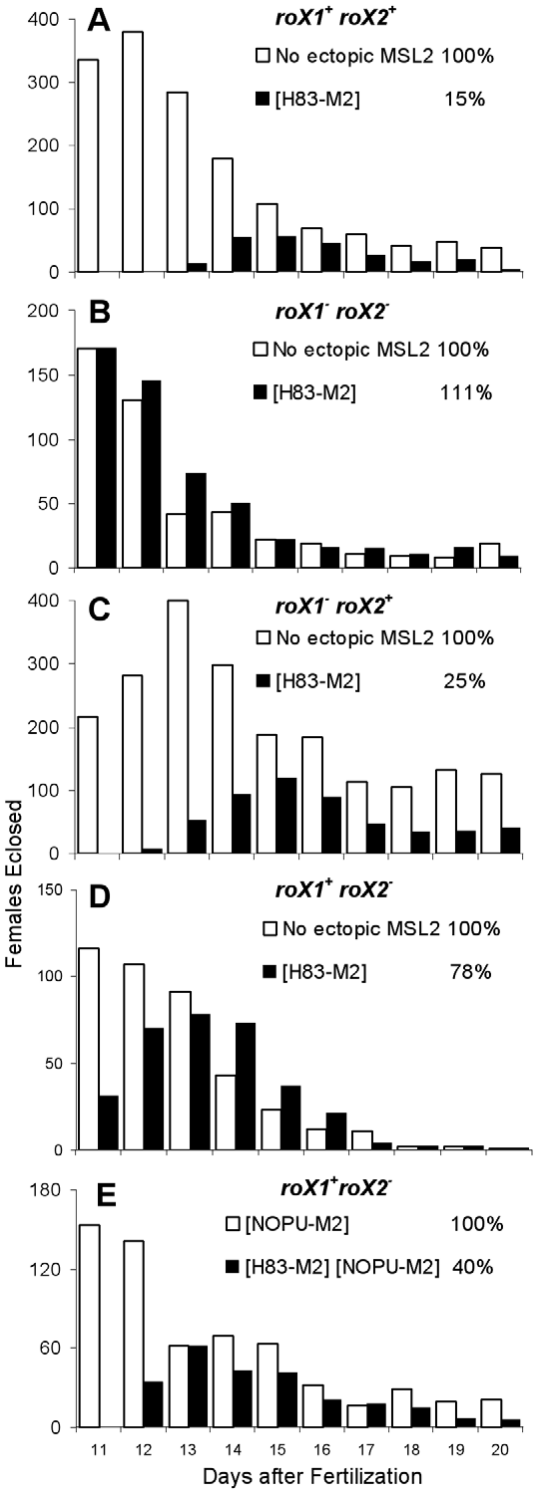
*roX2* mutant females escape the toxic effects of *H83M2*. Adult females eclosing each day were counted from a population of eggs laid on day one. White bars show non-transgenic females without ectopic MSL2 (except E), and black bars show females carrying the *H83M2* transgene. Each experiment varied the *roX* genotype. (A) *roX1^+^ roX2^+^*, (B) no *roX*, (C) *roX2^+^* only, (D) *roX1^+^* only, (E) *roX1^+^* only. Cumulative viability of *H83M2* females is shown as a percentage of their non-transgenic sisters.

Our data suggest that many cells lacking *roX2* RNA were unable to carry out dosage compensation because the remaining wild type *roX1* gene failed to switch on. If true, this should affect the viability of whole animals. As previously reported, ectopic MSL2 produced by *H83M2* is toxic to females (Figure 5A) [30] and toxicity requires *roX* RNA (Figure 5B) [17]. We found that *roX2* alone was almost equally toxic to females as when both RNAs were present (compare Figure 5A–5C). The surviving adults produced very few eggs and were sterile. However, *roX1* alone was much less toxic to females (Figure 5D) as would be expected if many cells lacked dosage compensation and thus escaped the toxic effects of MSL2 production. These surviving females were fertile. We conclude that the mosaic dosage compensation pattern is not limited to polytene tissues, but instead reflects a widespread failure of MSL2 protein made by the constitutive *H83M2* transgene to activate the endogenous *roX1* gene.

### *H83M2* does not turn on the expression of *roX1* reliably

Males that lack *roX2*, but are in all other ways wild type, have normal dosage compensation in all cells utilizing the remaining wild type *roX1* gene [17]. Finding spotty *roX1* activation only in animals relying on *H83M2* prompted us to examine why the transgene did not behave like the endogenous *msl2* locus in only this unusual genetic background. MSL2 made from the endogenous locus must activate *roX1* RNA production more effectively than transgenic MSL2. This was shown by introducing single wild type allele of endogenous *msl2* to *roX2*; *H83M2* males and observing dosage compensation in all cells (Figure S3G and S3I lane 5, Figure S4E and S4G lane 5). Next, we turned to the *NOPU-M2* transgene that also escapes SXL repression in females, but differs from *H83M2* by using the endogenous *msl2* promoter [21]. *NOPU-M2* is not toxic to females because it makes less MSL2 compared to *H83M2*. However, addition of the *NOPU-M2* transgene to *roX2* mutant females makes them sensitive to the toxic effects of *H83M2* (Figure 5E). Females carrying both *msl2* transgenes also drive activation of *roX1* expression in all cells (Figure S3H and S3I lane 6, Figure S4F and G lane 6). This is unlikely to be a simple additive effect of two pools of MSL2 protein since the *H83M2* transgene itself was fully capable of driving *roX1* expression in 100% of the cells when *roX2* was present (Figure S3C–S3D, and S3I lane 1–2 and Figure S4A–S4B and S4G lane 1–2). We propose that *H83M2* differs from the endogenous *msl2* gene in some way needed to reliably drive the *roX1* autoregulatory loop.

The mosaic pattern might arise from the *H83M2* transgene suffering from positional effect so MSL2 protein was not made in some cells. This is not the case since MSL2 expression can be clearly observed in all cells when both *roX* RNAs are present (Figure 4A). A second possibility is that *H83M2* initiates expression too late in embryogenesis to capture the early MSL-independent *roX1* transcripts we postulate are needed to begin the autoregulatory loop. We performed both MSL2 immunostaining and *roX1* FISH on *roX2*; *msl2*; *H83M2* embryos from 2 h to 20 h AEL. These embryos showed *H83M2* expression and subnuclear punctate *roX1* accumulation in essentially all cells at early gastrulation and remained on through at least until 20 hrs AEL when cuticle formation prevents reliable staining of internal tissues (Figure S5 and Figure S6). These results suggest that *H83M2* does come on during early embryogenesis and is effective in driving *roX1* transcription during the rapid cell divisions of embryonic development. However, many cells subsequently lose *roX1* transcription, and thus dosage compensation, later during larval stages.

### Cell cycle regulation of *msl2* expression

The MSL complex associates with many hundreds of actively transcribed genes along the male X. We considered the possibility that like core histone proteins, the MSL complex might need to abruptly increase its abundance following DNA replication. We set out to test if the transcription of *msl2* might be coupled to S phase.

**Figure 6:**
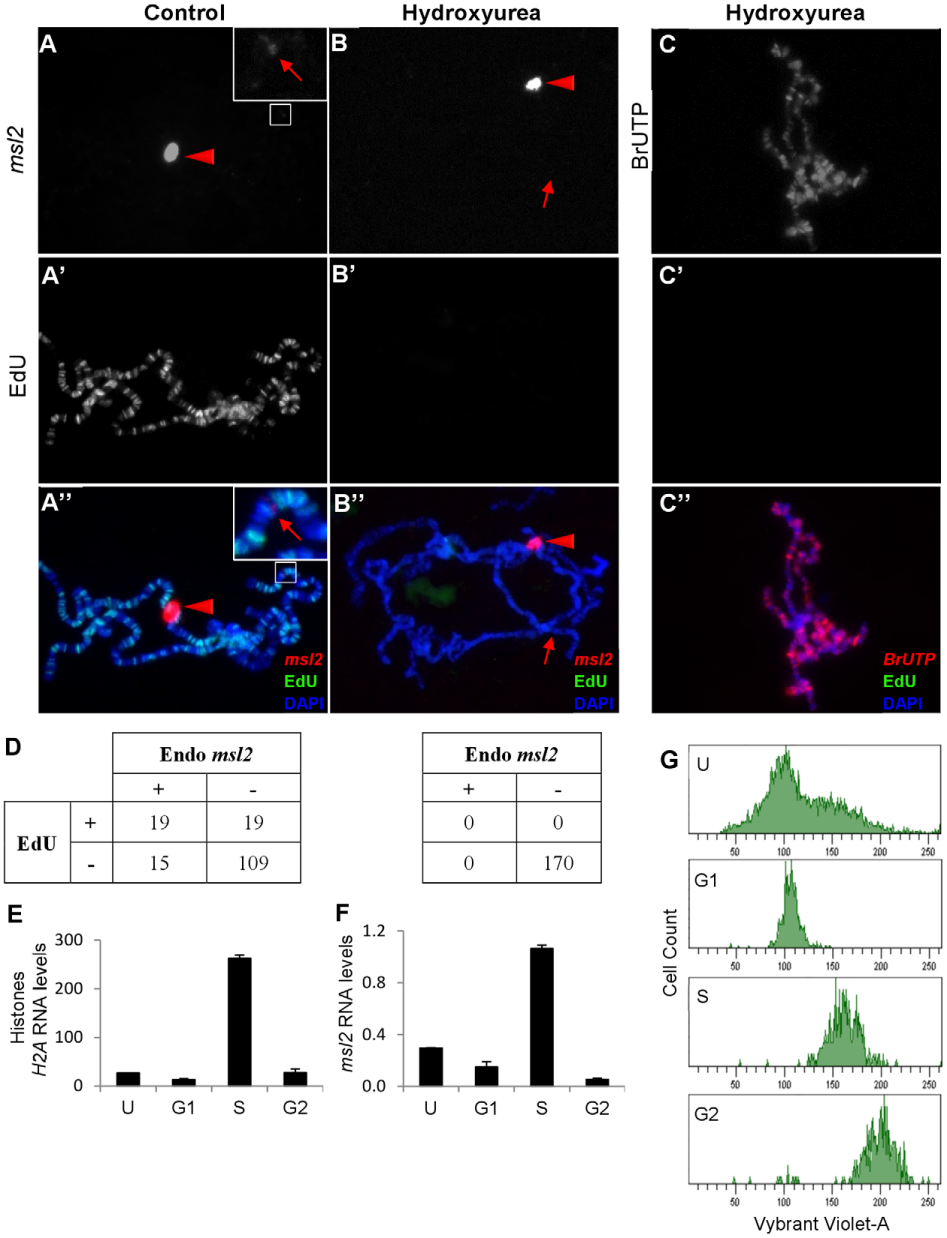
Transcription of *msl2* is correlated with cell cycle. Nascent *msl2* transcripts were detected with antisense FISH riboprobe in salivary glands. (A) Transgenic *H83M2* expression (bright signal indicated by red triangle) was observed at the 87A insertion site in all nuclei. Hybridization to the endogenous *msl2* locus at 23F (faint band indicated by red arrow in inset) was seen in only a minority of nuclei. Because of the difference in signal intensities between the two *msl2* loci, the inset is enhanced to show the weaker signal. (A′) EdU incorporation shows that this is one of the few nuclei undergoing endoreplication. (B) After treatment with 1 mM of HU (hydroxyurea), no cell transcribed the endogenous *msl2* gene (red arrow) but the transcription of *H83M2* continued (red triangle). (B′) HU blocked EdU incorporation from any cell. (C) Simultaneous treatment of salivary glands with HU, BrUTP, and EdU showed that blocking replication did not inhibit bulk transcription in these cells. (D) Many nuclei without (left table, N = 162) or with (right table, N = 170) HU treatment were scored for expression of endogenous *msl2* and EdU incorporation. Nascent transcripts were detected at the *H83M2* transgene in 100% of the nuclei (data not represented in the table). After sorting growing S2 cells into their respective phase of cell cycle via FACS, qPCR was done to quantify *H2A* (E) and *msl2* (F) transcripts levels normalized to *PKA*. (G) The FACS profile of unsorted (U) and sorted S2 cells (G1, S and G2 cell cycle). The sorted cells have a slightly higher content of Vybrant Violet-A dye because the cells are collected into tubes containing Vybrant Violet-A. The Y-axes are drawn on different scales.

We first used *in situ* hybridization to directly visualize actively growing *msl2* transcripts on polytene chromosomes. Most salivary gland cells have completed their endoreplication cycles at the wandering larval stage used in all our experiments, but a few cells are still actively replicating. When males carrying both the wild type endogenous *msl2* gene and the *H83M2* transgene were treated with antisense *msl2* riboprobes, all nuclei showed strong hybridization to a cloud of *msl2* RNA over the *H83M2* transgene inserted at 87A (Figure 6A). By contrast, a weaker hybridization signal was observed over the endogenous *msl2* gene at 23F in only about 20% of the nuclei. Most nuclei lacked detectable *msl2* transcripts at the endogenous locus. We tested whether the *H83M2* transgene might somehow inhibit expression of endogenous *msl2* gene by testing nontransgenic, wild type males. We found that again, only about 20% of male cells actively transcribed *msl2* (data not shown). This shows that while wild type males have abundant MSL2 protein painting the X chromosome in all cells, few cells are making new *msl2* mRNA.

To test if the sporadic transcription of endogenous *msl2* might coincide with the replication phase of the cell cycle, we briefly incubated dissected salivary glands in EdU to label newly replicated DNA, fixed the glands, and processed them for *msl2* FISH. We found a strong bias (Fisher's exact test p<0.0001) for replicating cells to also be transcribing *msl2*, but the overlap was not perfect (Figure 6A). For comparison we also examined *histone H2A* transcription, but even outside S phase every nucleus contained an intense hybridization signal over the endogenous *histone* gene cluster at polytene band 39DE (Figure S7). Only a portion of histone mRNA regulation occurs at transcription initiation. Most regulation is posttranscriptional [38], [39].

To directly test whether the sporadic *msl2* transcription pattern is associated with S phase, we treated our salivary glands with 1 mM of hydroxyurea (HU) to inhibit replication. EdU incorporation was completely blocked (Figure 6B′) but more significantly, transcripts were specifically lost only from the endogenous *msl2* locus at 23F while *msl2* transcripts made from the *H83M2* transgene at 87A remained strong (Figure 6B, 6B″). Likewise, HU treatment had no effect on bulk nascent transcripts as shown by strong incorporation of BrUTP on all chromosome arms (Figure 6C). These results show that *msl2* transcription normally occurs around S phase and is blocked by replication inhibitors. By contrast, *H83M2* does not show any cell cycle regulation. This difference might impact *roX1* expression.

To test whether the replication associated *msl2* transcription might be limited to only polytene cells with unusual cell cycles, we examined mitotically dividing diploid S2 cell in culture. Actively growing S2 cells were sorted into G1, S, and G2 (Figure 6G) via FACS (Fluorescence Activated Cell Sorting). RNA from these populations was assayed for *msl2*, *histone H2A*, and *PKA* transcripts by qPCR. As previously reported [38], *histone* RNA accumulates during S phase but is low in G1 and G2 (Figure 6E). Importantly, *msl2* transcripts are most abundant during S while lowest during G2 phase (Figure 6F). These results show that *msl2* RNA accumulation and synthesis normally occur predominantly in S phase, but the *H83M2* transgene lacks this coordination with replication.

We attempted to determine whether *roX1* transcription was also linked to S phase as might be expected, but were unsuccessful. We used both BrUTP and EU (Ethynyl Uridine) to label all new transcripts followed by a cold chase. We anticipated that the bulk of mRNA would leave the nucleus while newly labeled *roX1* RNA remained behind on the X. However, we were unable to detect any labeled RNA localized on the male X after chase (data not shown). We do not know if the signal was too weak to detect, or if the substitutions at the 5 position of uridine interferes with the function of RNA [40], leading to problems in *roX1* RNA folding, stability, MSL protein assembly, and/or targeting to the X.

## Discussion

Previous studies of *roX1* transcriptional control argued that either MSL2 alone or with a full set of MSL proteins was sufficient to drive male-specific expression [31]–[33]. Here, we present evidence that the expression of *roX1* gene is instead controlled through an autoregulatory loop. Pre-existing *roX* RNA, presumably in mature MSL complex, is required to drive new transcription. The reason we reached a different conclusion is largely attributed to removing the functionally redundant *roX2* in most of our experiments and assaying transcription only from the wild type *roX1* locus at its normal location on the X. The pathway we describe shares some elements with the negative regulatory loop between TFIIIA binding *5S* rDNA to drive transcription or 5S rRNA for storage during *Xenopus* oogenesis [41].

### A model for autoregulation of *roX1*

**Figure 7:**
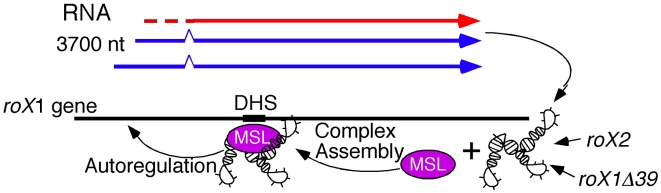
Autoregulation model. The earliest *roX1* transcripts (red) made at blastoderm originate from an uncharacterized MSL-independent promoter. This RNA may assemble with MSL protein subunits to produce the first functional MSL complexes needed to bind the internal DHS enhancer that drives sustained transcription (blue) from the male-specific promoters. When present, *roX2* RNA can also drive *roX1* transcription. Components of the replication pre-initiation complex also bind the DHS sequence in male cells (Figure S8A). The *msl2* transcripts are made predominantly during replication and new MSL2 protein is needed to assemble and stabilize newly made *roX1* RNA.

Figure 7 shows a model for how such an autoregulatory loop might operate. Because *roX* RNA is not maternally deposited into embryos, one problem is how male embryos could build their first MSL complex needed to initiate the cycle. Meller has shown that *roX1* transcription switches on in both sexes around blastoderm, just as general zygotic transcription begins [15], [16]. This suggests that an embryonic *roX1* promoter is active without MSL complex and could supply the first *roX1* RNA molecules to males, but these RNAs are eventually degraded in females. Early RNA assembles with MSL proteins and then drive *roX1* transcription from the known male-specific MSL-dependent promoters, setting up a positive autoregulatory loop necessary for the future maintenance of *roX1* expression in males. In our experiments, we showed that *roX2* RNA or truncated *roX1* RNA can also initiate endogenous *roX1* expression late in development after the early *roX1* transcripts are gone.

This model requires that male embryos preferentially sequester newly assembled MSL complex at the *roX1* gene to drive sustained transcription instead of allowing it to diffuse away to the vastly larger pool of ordinary X linked genes that must be dosage compensated. Only after *roX1* transcription is successfully upregulated can MSL complexes be released to the target genes along the X chromosome. Such behavior has been previously documented in cells containing abundant free MSL subunits and low levels of *roX1* transcription, exactly the conditions we believe occur as young male embryos initiate dosage compensation [13]. Examining cells shortly after MSL2 first turned on *roX1* transcription showed the earliest *roX1* transcripts remained near the site of synthesis (Figure 2B and 2C) consistent with the idea that newly formed MSL complexes preferentially act on the *roX1* gene. At later times, such as seen in *H83roX1-Δ39* animals that have had five days to drive endogenous *roX1* expression, every cell painted the entire X with *roX1* RNA (Figure 3G). While massive local cis spreading of the MSL complex has been reported for *roX1* and *roX2* transgenes inserted into autosomes [13], [18], [42], the physiological relevance of this is not widely accepted [25], [26], [43], [44]. Here we instead report striking local cis spreading of newly made MSL complex from the wild type *roX1* gene in its normal X chromosome environment (Figure S1E–S1F). Our data agree well with previous reports of local MSL spreading along the X [19] and support a role for cis spreading in the normal process of dosage compensation.

We have not directly determined what region of the *roX1* gene is necessary for autoregulation. However, a strong candidate is the ∼200 bp male-specific DNase I hypersensitive site (DHS) found about 1.5 kb downstream of the adult *roX1* promoters. The DHS is sufficient to recruit the MSL complex to ectopic sites when moved to the autosomes in a sequence specific manner [45], [46]. While partial complexes lacking MOF or MSL3 remain bound to the DHS, incomplete complexes lacking either *roX* RNA or the MLE RNA helicase postulated to fold *roX* RNA bind the DHS poorly [45], [46]. This element stimulates *roX1* transcription when MSL complex is bound and represses basal transcription when MSL complex is absent. Also, deleting the DHS greatly reduces transcription of *roX1* transgenes [33]. Together, these findings support a model where transcription from the *roX1* gene requires pre-existing *roX* RNA within MSL complexes bound to the internal DHS enhancer. However, this view is likely an oversimplification because very large internal deletions such as *roX1* ^ex7B^, comparable to our *H83roX1-Δ39* transgene (Figure 3A), remain transcriptionally active despite the loss of the DHS enhancer [32], [47].

### Cell cycle regulation

Translation of *msl2* mRNA is normally subject to elaborate controls acting through the 5′ and 3′ UTRs [48]–[52]. Little attention has been given to its transcription control, although recently anti-MSL2 antibodies were found to precipitate *msl2* mRNA [53]. We found that *msl2* transcription is associated with replication, and this is likely important for its normal control. Without MSL2 protein, naked *roX1* RNA is rapidly destroyed [11]. Here we found the converse; cells rapidly clear any MSL2 protein not bound to *roX* RNA. When free MSL2 subunits are artificially stabilized with proteosome inhibitors, they coat all the chromosomes indiscriminately. This implies that the synthesis of MSL2 and *roX* RNAs are closely coordinated so each component stabilizes the other ensuring that only correctly targeted molecules survive. Although MSL2 lacks known RNA binding motifs, previous work of others is consistent with an intimate interaction between MSL2 and *roX* RNA [31]. We suspect the loss of replication-coupled transcription may contribute to the failure of dosage compensation in some cells relying exclusively on *H83M2* for MSL2 protein and *roX1* for *roX* RNA. This defect is corrected when both *roX1* and MSL2 are coordinately made from the same *hsp83* promoter (Figure 3G). Cells in mosaic animals lose dosage compensation sometime between the end of embryogenesis and third instar larvae. Many tissues undergo significant changes in cell cycle near the end of embryogenesis, particularly the introduction of G1 [54], and we suspect this shift contributes to loss of dosage compensation in our mosaic animals. If MSL complex should ever drop below the level needed to sustain the autoregulatory *roX1* loop, it could never recover regardless of later MSL2 production. The details remain unclear because *H83M2* is vigorously transcribed at the developmental times we examined, including S phase. We do not know whether the regulatory *msl2* UTR sequences removed from *H83M2* disrupt additional posttranscriptional controls that might promote efficient translation during replication.

A second issue is that the X is painted with MSL complex throughout the cell cycle. This might drive continuous rather than cyclic *roX1* synthesis. While we were unable to directly determine if *roX1* transcription is cell cycle regulated, we note that the replication machinery components ORC2 and MCM are bound specifically to the *roX1* DHS enhancer only in male cells (Figure S8A). The significance of this is not known, but it is tempting to speculate that components of the pre-initiation complex bound to DHS compete with MSL complex thus inhibiting *roX1* transcription in G1. Firing of the replication origins removes ORC2 and MCM possibly allowing MSL complex access to the DHS and so switch on transcription shortly after the onset of S phase. The replication machinery is commonly found near the promoters of many genes [55], including *msl2* (Figure S8B), so further experiments will be needed to determine if such binding actually plays any regulatory role here.

### Regulation of *roX2*

While we did not specifically address transcriptional control of *roX2*, it must differ from *roX1* in several important ways. Meller has previously shown that *roX2* transcription lags *roX1* by a few hours during early embryonic development and is always limited to males [16]. Here we found that *roX2* transcription differs by not requiring pre-existing *roX* RNA and can be switched on several days later during larval development simply by ectopically expressing MSL2. MSL2 protein made by the constitutive *H83M2* transgene only sporadically activates *roX1*, but robustly drives *roX2*. The *roX2* gene also carries a DHS enhancer similar to that found in *roX1* [45], but if it plays a comparable role in *roX2* regulation, it presumably would not require complete MSL complex. The region near the proline rich domain towards the C-terminus of MSL2 is essential for this regulation [31].

### Parallels between *roX1* and *Sxl*

The *roX1* autoregulation loop described above shares parallels to the SXL autoregulatory loop controlling all aspects of sex determination and dosage compensation in *Drosophila*. X∶A counting elements act upon an early establishment *Sxl* promoter to make SXL in early female embryos. These first SXL proteins stimulate productive splicing of *Sxl* mRNAs transcribed from a distinct maintenance promoter ensuring further SXL production [56]. MSL1, MSL3, MOF, and MLE are unable to package early *roX1* RNA made from the embryonic promoter in female embryos to form a fully functional mature MSL complex. To do that, MSL2 protein made only in males is required. We suspect these earliest MSL complexes sustain *roX1* transcription as embryos switch to the MSL-dependent promoters. Recently a new role for MSL2 in females has been described during the brief window when *Sxl* autoregulation is established [57]. Perhaps females also fleetingly utilize the early burst of *roX1* before abundant SXL represses *msl2* translation.

Thousands of large noncoding RNAs have recently been discovered in vertebrates [2], [58], many of which are associated with chromatin remodeling enzymes [1], [59]–[61]. It is likely that some of these will face similar regulatory and functional demands as the *roX* RNAs and may have evolved comparable strategies to control their production. For instance, the short RepA sequence at the 5′ end of mammalian *Xist* RNA may influence production of full length transcripts [59].

## Materials and Methods

### *Drosophila* stocks

Larvae and flies were raised on standard cornmeal-yeast-agar-molasses medium containing propanoic acid at 25°C. In all experiments the *roX1* mutation is *roX1^ex6^* and the *roX2* allele is *Df (1) roX2^52^* [17]. The *[w^+^ 4Δ4.3]* transgene supplies essential adjacent genes lost in the *roX2* deletion.

The transgenic flies used have been previously described as follows: [*w* ^+^ *H83M2-6I*] [30], [*w* ^+^ *NOPU-M2*] [*21*], and [*w* ^+^ *H83roX1-Δ39*] [*13*]. The *H83M2* transgene was recombined with *[P{SUPor-P}KG02776]* which contains *y^+^* as a marker so larvae of the correct genotype could be recognized by mouth hook color (Flybase). The *P{GAL4-Act5C(FRT.CD2).P}*, *P{UAS-GFP.S65T}* and *MKRS-hsFLP* transgenic fly stocks were provided by Graeme Mardon.

The *UASp-MSL2* transgene was made by digesting the *H83M2* transgene with *EcoRI* and subcloning the MSL2 ORF into the *pUAS-P2* plamid vector (a gift from Pernille Rørth).

### Heatshock

The female larvae for the flp-out experiment were kept at 25°C and heatshocked at 37°C for 1 hr each at day 4 and 5 AEL. Their salivary glands were then dissected on day 6 AEL, fixed and immunostained as described below.

The full genotypes of the larvae heatshocked in Figure 1 are as follows:

B-F) *y w; [w^+^ y^+^Act-FRT-CDC2-FRT-Gal4] [w^+^UAS-GFP] +/+ + [w^+^UASp-MSL2]; MRKS [Hs-Flp]/+*

H-L) *y w roX2^52^ [w^+^4Δ4.3]; [w^+^ y^+^Act-FRT-CDC2-FRT-Gal4] [w^+^UAS-GFP] +/+ + [w^+^UASp-MSL2]; MRKS [Hs-flp]/+*

The full genotypes of the larvae in Figure 2 are identical to Figure 1H–1L.

The full genotypes of the larvae in Figure 3 are as follows:

A) *y w roX2^52^ [w^+^4Δ4.3]; msl2; [w^+^H83-M2] [w^+^y^+^P{SUPor-P}KG02776]*/+ +

B) *y w roX2^52^ [w^+^4Δ4.3]/Y; msl2; [w^+^H83-M2] [w^+^y^+^P{SUPor-P}KG02776]*/+ +

D) *y w/Y*

E) *y w*

F) *y w roX2^52^ [w^+^4Δ4.3]/Y; msl2; [w^+^H83-M2] [w^+^y^+^ P{SUPor-P}KG02776]/+ +*

G) *y w roX2^52^ [w^+^4Δ4.3]/Y; msl2; [w^+^H83-M2] [w^+^H83-roX1Δ39] [w^+^y^+^ P{SUPor-P}KG02776]/+ + +*

H) *y w roX1^ex6^roX2^52^ [w^+^4Δ4.3]/Y; msl2; [w^+^H83-M2] [w^+^H83-roX1Δ39] [w^+^y^+^ P{SUPor-P}KG02776]/+ + +*

The full genotypes of the larvae in Figure 4 are as follows:

A-A″) *y w; msl2; [w^+^H83-M2] [w^+^y^+^ P{SUPor-P}KG02776]*

B-B″) *y w roX2^52^ [w^+^4Δ4.3]/Y; msl2; [w^+^H83-M2] [w^+^y^+^P{SUPor-P}KG02776]*/+ +

C-C″) *y w roX1^ex6^ roX2^52^ [w^+^4Δ4.3]/Y; msl2/*+; *[w^+^H83-M2] [w^+^y^+^P{SUPor-P}KG02776]/+ +*

D-D″) Same as C

The full genotypes of the parents in the crosses for Figure 5A–5E are as follows:

A) *y w/Y; msl2; [w^+^H83-M2] [w^+^y^+^ P{SUPor-P}KG02776]/+ +* X *y w; msl2*

B) *y w roX1^ex6^/Y; msl2; [w^+^H83-M2] [w^+^y^+^ P{SUPor-P}KG02776]*/+ + X *y w roX1^ex6^; msl2*

C) *y w roX2^52^ [w^+^4Δ4.3]/Y; msl2; [w^+^H83-M2] [w^+^y^+^ P{SUPor-P}KG02776]*/+ + X *y w roX2^52^ [w^+^4Δ4.3]; msl2*

D) *y w roX1^ex6^ roX2^52^ [w^+^4Δ4.3]/Y; msl2/*+; *[w^+^H83-M2] [w^+^y^+^ P{SUPor-P}KG02776] +/+ + [w^+^GMroX1^+^]* X *y w roX1^ex6^ roX2^52^ [w^+^4Δ4.3]*

E) *y w roX2^52^ [w^+^4Δ4.3]/Y; msl2; [w^+^H83-M2] [w^+^y^+^ P{SUPor-P}KG02776]*/+ + X *y w roX2^52^ [w^+^4Δ4.3]; [w^+^NOPU-M2]*

### Immunohistochemistry and *in situ* hybridization

Immunostaining and *in situ* hybridization on third instar salivary glands was performed as described in [11] except for the following modification. The slides were treated with proteinase K (20 µg/ml in PBT) for 3 min. Each slide was hybridized with 5 ng of *roX1*, *msl2* or *H2A* biotin-labeled single-stranded antisense riboprobes using the T7 high yield Transcription kit #K0441 (Fermentas). Fluorescent development was done as instructed by the Tyramide Signal Amplification (TSA) kit (NEL700A) (PerkinElmer) with SA-FITC (NEL720) (PerkinElmer) or SA-Texas Red (NEL 721) (PerkinElmer). For double ISH, we labeled the second antisense riboprobes with digoxygenin. Before adding the anti-digoxygenin-HRP conjugate, the first horseradish peroxidase conjugate was quenched in 3% H_2_O_2_ for 30 mins. The second color is then developed using Tyramide-Alexa488 (T20922) (Invitrogen). To inhibit MSL2 degradation, we incubate imaginal discs and salivary glands with 10 µM of MG132 (474791) (Calbiochem) dissolved in Schneider Culture Media for 3 hours before proceeding with immunostaining.

### EdU labeling

To block replication, salivary glands from 3^rd^ instar larvae were first incubated in 1 mM HU for 1 h. If not, they were simply incubated in S2 cell culture media (see below). After which, the glands were transferred and incubated in EdU for 15 minutes followed immediately by fixation. The detection was then performed as instructed by the Click-iT EdU imaging kit #C10337 (Invitrogen). Both HU and EdU were dissolved in S2 cell culture media before use.

### Co-labeling replicating DNA and nascent transcripts via EdU and BrUTP respectively

Salivary glands were initially treated the same way as described above. However, after HU treatment, the glands were incubated in a BrUTP/DOTAP/EdU mixture instead for 15 mins. The BrUTP/DOTAP mixture was previously described to allow efficient nucleotide triphosphate uptake by the cell to label nascent transcripts [62]. EdU detection was performed as described above. Rat Anti-BrdU monoclonal antibodies #NB500-169 (Novus Biological) and Goat anti-rat Alexa 594 #A11007 (Invitrogen) were then used as primary and secondary antibodies respectively, to detect BrUTP.

### Cell culture and FACS

Schneider 2 (S2) cells were cultured in 15-cm plates at a density of 1×10^6^ cells/ml in Schneider's Media #11720034 (Invitrogen) supplemented with 10% FCS and penicillin/streptomycin. After reaching a density of 5×10^6^ cells/ml, the cells were re-suspended in new culture media containing 5 µM of Vybrant Dye Cycle Violet #V35003 (Invitrogen) to stain the DNA. The cells were then sorted into their respective phase of cell cycle via FACSAria II (BD Biosciences) at the Cytometry and Cell Sorting Facility located in Baylor College of Medicine.

### Real-time PCR

Total RNA was extracted from cells using Trizol® reagent 15596-018 (Invitrogen) as per the manufacturer's protocol. cDNA was synthesized from 0.5–1 µg of total RNA using random hexamers and MMLV Reverse transcriptase #M1701 (Promega) as per manufacturer's protocol. The cDNA was purified using MinElute PCR kit #28004 (Qiagen).

Real-time PCR was performed using an Applied Biosystems 7300 Sequence Detection system. The 25 µl PCR included 1 µl cDNA, 1× SYBR® Green PCR Master Mix #4309155 (Applied Biosystems) and 1 µl of gene specifc primers. The reactions were incubated in a 96-well optical plate at 95°C for 10 min, followed by 40 cycles of 95°C for 15 s and 60° for 10 min. The Ct data was determinate using default threshold settings. The threshold cycle (Ct) is defined as the fractional cycle number at which the fluorescence passes the fixed threshold. PKA is used as an endogenous standard for normalization of *histone H2A* and *msl2*. The primers used for qPCR are: H2A forward TGGACGTGGAAAAGGTGGCA; H2A reverse ACGGCAGCTAGGTAAACTGGAG; MSL2 forward GGCGAGTACCAGGGCTTCAATATC; MSL2 Reverse TGCTGCAGCTGGACACGAATAG; PKA forward – AGCCGCACTCGCGCTTCTAC and PKA reverse - AGCCGGAGAATCTGCTGATTG.

## Supporting Information

Figure S1 The *[H83-roX1Δ39]* transgene turns on endogenous *roX1* in males. (A) *roX1* transcripts (Orange) and antisense probes (green). (B) Quantification of *roX1* hybridization over polytene X chromosomes: entire X (black), only distal X (gray, example E), a single band at the *roX1* locus (hatched, example F red arrowhead), or no staining (white). N = nuclei counted. The *[H83-Δ39roX1]* transgene does not have an effect on females. (C) The internal probe does not hybridize to *Δ39roX1* RNA, (D) but the *roX1Δ39* probe does. White line delineates the X chromosome. (E) Males display a diverse pattern of *roX* painting, ranging from local spreading from the *roX1* locus to (F) just a single band (indicated by the red arrow). (G) *Δ39roX1* RNA (not visualized) helps *[H83-M2]* strongly activate endogenous *roX1* (detected with internal probe) in almost all cells (two nuclei shown) (H) Similar *roX1* staining was found in all squashed imaginal disc cells.
(PDF)

Figure S2 MSL immunostaining reveals that MSL2 binds indiscriminately to all the chromosomes in the absence of any roX RNA. (A) In the presence of *roX* RNA, MSL2 binds and paints the X chromosome only. (B) In the absence of *roX* RNA, the MSL proteins form incomplete complexes and binds to all the chromosomes, albeit poorly. (C) After treatment with MG132, a proteasome inhibitor, strong MSL2 binding can now be observed to occur throughout the nucleus. *roX1^−^ roX2^−^* males are sick and do not survive till adulthood. However, sick and rare 3^rd^ instar larvae can be obtained for salivary squashes although the chromosomes have extremely poor morphology and easily shattered during squashes. (D) The same experiment was repeated in whole mount salivary glands and MSL2 can be seen concentrated on the X chromosome. (E) At low resolution, MSL2 staining becomes undetectable in the absence of *roX* RNA (compared to B). (F) MSL2 can be seen binding to the entire nucleus when degradation is being inhibited.
(PDF)

Figure S3 MSL immunostaining of polytene chromosomes in *[H83-M2]* expressing larvae reveals mosaic establishment in DC. MSL immunostaining and DAPI is represented by red and blue respectively. (A) The MSL complex is bound along the male single X chromosome at hundreds of bands. (B) Due to the lack of MSL2, female do not have MSL binding to the X chromosome. (C–D) Painting of the X can be restored by the *[H83-M2]* transgene in *msl2* mutant animals if both *roX1* and *roX2* are present. (E) The MSL complex fails to paint the entire X chromosome in males if *roX2* is deleted. This cis-spreading phenomenon around the *roX1* locus (indicated by red arrow) is similar to the autosomal spreading of *roX1* transgene observed under low transcription rate (*9*). (F) In females, the X chromosome is either painted (arrowhead) or not painted (arrow). See Figure 1I for quantification. (G–H) Normal painting of the X is re-established when an endogenous copy of *msl2* is restored. In females, this is achieved by co-expressing the *[NOPU-M2]* transgene. (I) The fraction of polytene nuclei displaying complete, partial, or no X MSL1 painting is shown. N = nuclei scored. Genotypes: (1–2) *roX1^+^roX2^+^;msl2;[H83-M2]*/+, (3–4) *roX1^+^roX2^−^;msl2;[H83-M2]*/+, (5) *roX1^+^roX2^−^/Y;msl2/+;[H83-M2]*/+, (6) *roX1^+^roX2^−^;msl2/+;[NOPU-M2] [H83-M2]*/+.
(PDF)

Figure S4 MSL2 immunostaining of imaginal disc cells in *[H83-M2]* expressing larvae reveals mosaic establishment of DC. (A–B) Painting in all nuclei is observed in animals relying upon the *[H83-M2]* transgene if both *roX1* and *roX2* are present. (C–D) Mosaic painting of the X chromosome is observed in both males and females when *roX2* is deleted. White arrows indicated unpainted nucleus. (E–F) Normal painting of the X is re-established when an endogenous copy of *msl2* is restored. In females, this is achieved by co-expressing the *[NOPU-M2]* transgene. (G) Cells with (black) and without (white) obvious subnuclear domain MSL2 staining taken from gently squashed discs were counted (Figure S1) N = cells counted. Genotypes: (1–2) *roX1^+^roX2^+^;msl2;[H83-M2]*/+, (3–4) *roX1^+^roX2^−^;msl2;[H83-M2]*/+, (5) *roX1^+^roX2^−^/*Y;*msl2/+;[H83-M2]*/+ male, (6) *roX1^+^roX2^−^;msl2/+; [NOPU-M2][H83-M2]*/+ female.
(PDF)

Figure S5 MSL2 immunostaining of embryos reveals that 100% of the cells expressed *[H83-MSL2]*. (A–A″) A wildtype embryo at the end of germband extension showing endogenous MSL2 expression detected in 100% of the cells (B–B″) A *roX2^−^; msl2; [H83M2]* embryos, expected to display a mosaic pattern of dosage compensation in imaginal discs and salivary glands by 3^rd^ instar, shows no signs of mosaicism during early embryogenesis.
(PDF)

Figure S6 *roX1 in situ* hybridization of embryos reveals that 100% of the cells successfully establish dosage compensation. (A–A″) A wildtype embryo at about 20 hr AEL, has *roX1* expression detected in 100% of the cells (B–B″) A similarly stage *roX2^−^; msl2; [H83M2]* embryos displaying no signs of mosaicism.
(PDF)

Figure S7 Histones transcripts not a good marker of S Phase. (A–A″) Nascent *H2A* (red arrow) and *msl2* transcripts (red arrowhead) can be visualized at cytolocation 23F and 39 respectively via double ISH of polytene chromosomes with anti-sense *msl2* and *H2A* RNA probes. (B) Although it is well documented that the transcription rate of histones decreases by 5 folds upon Hydroxurea treatment [38], it is hard to quantified using the TSA technique for ISH. (B′–B″) On the other hand, *msl2* transcripts have completely disappeared upon HU treatment.
(PDF)

Figure S8 Components of the DNA replication preinitiation complex bind transcriptional control regions within both *roX1* and *msl2* genes. (A) Both MCM and ORC2 bind the DHS control region within *roX1* only in male cells. (B) Preinitiation complex is located near the promoters of many genes including *msl2*. Binding data shown for S2 cells. Screen captures of ChIP-seq data of the MacAlpine lab deposited in FlyBase Modencode. Y-axes not the same for different profiles.
(PDF)

## References

[1] Khalil AM, Guttman M, Huarte M, Garber M, Raj A (2009). Many human large intergenic noncoding RNAs associate with chromatin-modifying complexes and affect gene expression.. Proc Natl Acad Sci U S A.

[2] Orom UA, Derrien T, Beringer M, Gumireddy K, Gardini A (2010). Long noncoding RNAs with enhancer-like function in human cells.. Cell.

[3] Deng X, Meller VH (2006). Non-coding RNA in fly dosage compensation.. Trends Biochem Sci.

[4] Gelbart M, Kuroda M (2009). Drosophila dosage compensation: a complex voyage to the X chromosome.. Development.

[5] Hilfiker A, Hilfiker-Kleiner D, Pannuti A, Lucchesi JC (1997). *mof*, a putative acetyl transferase gene related to the *Tip60* and *MOZ* human genes and to the *SAS* genes of yeast, is required for dosage compensation in Drosophila.. EMBO J.

[6] Akhtar A, Becker PB (2000). Activation of transcription through histone H4 acetylation by MOF, an acetyltransferase essential for dosage compensation in Drosophila.. Mol Cell.

[7] Bone JR, Lavender J, Richman R, Palmer MJ, Turner BM (1994). Acetylated histone H4 on the male X chromosome is associated with dosage compensation in Drosophila.. Genes Dev.

[8] Smith ER, Allis CD, Lucchesi JC (2001). Linking global histone acetylation to the transcription enhancement of X-chromosomal genes in Drosophila males.. J Biol Chem.

[9] Wu L, Zee BM, Wang Y, Garcia BA, Dou Y (2011). The RING Finger Protein MSL2 in the MOF Complex Is an E3 Ubiquitin Ligase for H2B K34 and Is Involved in Crosstalk with H3 K4 and K79 Methylation.. Mol Cell.

[10] Amrein H, Axel R (1997). Genes expressed in neurons of adult male Drosophila.. Cell.

[11] Meller VH, Gordadze PR, Park Y, Chu X, Stuckenholz C (2000). Ordered assembly of *roX* RNAs into MSL complexes on the dosage-compensated X chromosome in Drosophila.. Curr Biol.

[12] Franke A, Baker BS (1999). The *roX1* and *roX2* RNAs are essential components of the compensasome, which mediates dosage compensation in Drosophila.. Mol Cell.

[13] Kelley RL, Lee OK, Shim YK (2008). Transcription rate of noncoding *roX1* RNA controls local spreading of the Drosophila MSL chromatin remodeling complex.. Mech Dev.

[14] Park SW, Kang Y, Sypula JG, Choi J, Oh H (2007). An evolutionarily conserved domain of *roX2* RNA is sufficient for induction of H4-Lys16 acetylation on the Drosophila X chromosome.. Genetics.

[15] Meller VH, Wu KH, Roman G, Kuroda MI, Davis RL (1997). *roX1* RNA paints the X chromosome of male Drosophila and is regulated by the dosage compensation system.. Cell.

[16] Meller VH (2003). Initiation of dosage compensation in Drosophila embryos depends on expression of the *roX* RNAs.. Mech Dev.

[17] Meller VH, Rattner BP (2002). The *roX* genes encode redundant male-specific lethal transcripts required for targeting of the MSL complex.. EMBO J.

[18] Park Y, Kelley RL, Oh H, Kuroda MI, Meller VH (2002). Extent of chromatin spreading determined by *roX* RNA recruitment of MSL proteins.. Science.

[19] Oh H, Park Y, Kuroda MI (2003). Local spreading of MSL complexes from *roX* genes on the Drosophila X chromosome.. Genes Dev.

[20] Bashaw GJ, Baker BS (1997). The regulation of the Drosophila *msl-2* gene reveals a function for Sex-lethal in translational control.. Cell.

[21] Kelley RL, Wang J, Bell L, Kuroda MI (1997). Sex lethal controls dosage compensation in Drosophila by a non-splicing mechanism.. Nature.

[22] Fauth T, Muller-Planitz F, Konig C, Straub T, Becker PB (2010). The DNA binding CXC domain of MSL2 is required for faithful targeting the Dosage Compensation Complex to the X chromosome.. Nucleic Acids Res.

[23] Bhadra U, Pal-Bhadra M, Birchler JA (1999). Role of the *male specific lethal* (*msl*) genes in modifying the effects of sex chromosomal dosage in Drosophila.. Genetics.

[24] Bhadra U, Pal-Bhadra M, Birchler JA (2000). Histone acetylation and gene expression analysis of *Sex lethal* mutants in Drosophila.. Genetics.

[25] Dahlsveen IK, Gilfillan GD, Shelest VI, Lamm R, Becker P (2006). Targeting Determinants of Dosage Compensation in Drosophila.. PLoS Genet.

[26] Gilfillan GD, Konig C, Dahlsveen IK, Prakoura N, Straub T (2007). Cumulative contributions of weak DNA determinants to targeting the Drosophila dosage compensation complex.. Nucleic Acids Res.

[27] Gu W, Szauter P, Lucchesi JC (1998). Targeting of MOF, a putative histone acetyl transferase, to the X chromosome of Drosophila melanogaster.. Dev Genet.

[28] Kind J, Akhtar A (2007). Cotranscriptional recruitment of the dosage compensation complex to X-linked target genes.. Genes Dev.

[29] Lyman LM, Copps K, Rastelli L, Kelley RL, Kuroda MI (1997). Drosophila male-specific lethal-2 protein: structure/function analysis and dependence on MSL-1 for chromosome association.. Genetics.

[30] Kelley RL, Solovyeva I, Lyman LM, Richman R, Solovyev V (1995). Expression of *msl-2* causes assembly of dosage compensation regulators on the X chromosomes and female lethality in Drosophila.. Cell.

[31] Li F, Schiemann AH, Scott MJ (2008). Incorporation of the noncoding *roX* RNAs alters the chromatin-binding specificity of the Drosophila MSL1/MSL2 complex.. Mol Cell Biol.

[32] Rattner BP, Meller VH (2004). Drosophila male-specific lethal 2 protein controls sex-specific expression of the *roX* genes.. Genetics.

[33] Bai X, Alekseyenko AA, Kuroda MI (2004). Sequence-specific targeting of MSL complex regulates transcription of the *roX* RNA genes.. EMBO J.

[34] Franke A, Dernburg A, Bashaw GJ, Baker BS (1996). Evidence that MSL-mediated dosage compensation in Drosophila begins at blastoderm.. Development.

[35] Xu T, Harrison SD (1994). Mosaic analysis using FLP recombinase.. Methods Cell Biol.

[36] Chang KA, Kuroda MI (1998). Modulation of MSL1 abundance in female Drosophila contributes to the sex specificity of dosage compensation.. Genetics.

[37] Palmer MJ, Richman R, Richter L, Kuroda MI (1994). Sex-specific regulation of the *male-specific lethal-1* dosage compensation gene in Drosophila.. Genes Dev.

[38] Sittman DB, Graves RA, Marzluff WF (1983). Histone mRNA concentrations are regulated at the level of transcription and mRNA degradation.. Proc Natl Acad Sci U S A.

[39] Marzluff WF, Wagner EJ, Duronio RJ (2008). Metabolism and regulation of canonical histone mRNAs: life without a poly(A) tail.. Nat Rev Genet.

[40] Schmittgen TD, Danenberg KD, Horikoshi T, Lenz HJ, Danenberg PV (1994). Effect of 5-fluoro- and 5-bromouracil substitution on the translation of human thymidylate synthase mRNA.. J Biol Chem.

[41] Wolffe AP, Brown DD (1988). Developmental regulation of two 5S ribosomal RNA genes.. Science.

[42] Larschan E, Alekseyenko AA, Gortchakov AA, Peng S, Li B (2007). MSL Complex Is Attracted to Genes Marked by H3K36 Trimethylation Using a Sequence-Independent Mechanism.. Mol Cell.

[43] Gilfillan GD, Straub T, de Wit E, Greil F, Lamm R (2006). Chromosome-wide gene-specific targeting of the Drosophila dosage compensation complex.. Genes Dev.

[44] Fagegaltier D, Baker BS (2004). X Chromosome Sites Autonomously Recruit the Dosage Compensation Complex in Drosophila Males.. PLoS Biol.

[45] Park Y, Mengus G, Bai X, Kageyama Y, Meller VH (2003). Sequence-specific targeting of Drosophila *roX* genes by the MSL dosage compensation complex.. Mol Cell.

[46] Kageyama Y, Mengus G, Gilfillan G, Kennedy HG, Stuckenholz C (2001). Association and spreading of the Drosophila dosage compensation complex from a discrete *roX1* chromatin entry site.. EMBO J.

[47] Deng X, Rattner BP, Souter S, Meller VH (2005). The severity of *roX1* mutations is predicted by MSL localization on the X chromosome.. Mech Dev.

[48] Medenbach J, Seiler M, Hentze MW (2011). Translational Control via Protein-Regulated Upstream Open Reading Frames.. Cell.

[49] Duncan K, Grskovic M, Strein C, Beckmann K, Niggeweg R (2006). Sex-lethal imparts a sex-specific function to UNR by recruiting it to the *msl-2* mRNA 3′ UTR: translational repression for dosage compensation.. Genes Dev.

[50] Grskovic M, Hentze MW, Gebauer F (2003). A co-repressor assembly nucleated by Sex-lethal in the 3′UTR mediates translational control of Drosophila *msl-2* mRNA.. EMBO J.

[51] Gebauer F, Grskovic M, Hentze MW (2003). Drosophila Sex-lethal inhibits the stable association of the 40S ribosomal subunit with *msl-2* mRNA.. Mol Cell.

[52] Gebauer F, Corona DF, Preiss T, Becker PB, Hentze MW (1999). Translational control of dosage compensation in Drosophila by Sex-lethal: cooperative silencing via the 5′ and 3′ UTRs of *msl-2* mRNA is independent of the poly(A) tail.. EMBO J.

[53] Johansson AM, Allgardsson A, Stenberg P, Larsson J (2011). *msl2* mRNA is bound by free nuclear MSL complex in Drosophila melanogaster.. Nucleic Acids Res.

[54] Du W, Dyson N (1999). The role of RBF in the introduction of G1 regulation during Drosophila embryogenesis.. EMBO J.

[55] MacAlpine HK, Gordan R, Powell SK, Hartemink AJ, MacAlpine DM (2010). Drosophila ORC localizes to open chromatin and marks sites of cohesin complex loading.. Genome Res.

[56] Cline TW, Meyer BJ (1996). Vive la difference: males vs females in flies vs worms.. Annu Rev Genet.

[57] Gladstein N, McKeon MN, Horabin JI (2010). Requirement of male-specific dosage compensation in Drosophila females–implications of early X chromosome gene expression.. PLoS Genet.

[58] Guttman M, Amit I, Garber M, French C, Lin MF (2009). Chromatin signature reveals over a thousand highly conserved large non-coding RNAs in mammals.. Nature.

[59] Zhao J, Sun BK, Erwin JA, Song JJ, Lee JT (2008). Polycomb proteins targeted by a short repeat RNA to the mouse X chromosome.. Science.

[60] Mohammad F, Mondal T, Guseva N, Pandey GK, Kanduri C (2010). *Kcnq1ot1* noncoding RNA mediates transcriptional gene silencing by interacting with Dnmt1.. Development.

[61] Nagano T, Mitchell JA, Sanz LA, Pauler FM, Ferguson-Smith AC (2008). The *Air* noncoding RNA epigenetically silences transcription by targeting G9a to chromatin.. Science.

[62] Chang WY, Winegarden NA, Paraiso JP, Stevens ML, Westwood JT (2000). Visualization of nascent transcripts on Drosophila polytene chromosomes using BrUTP incorporation.. Biotechniques.

