# Supplementary figures and images for: Autoregulation of the Drosophila Noncoding roX1 RNA Gene
Source: PLoS Genet. 2012 Mar 15;8(3):e1002564. doi: 10.1371/journal.pgen.1002564 (PMC3305356; doi:10.1371/journal.pgen.1002564)

**A**

Endo *roX1* = 3.7 KB 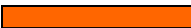  
 [*H83-roX1Δ39*] = 1.2 KB 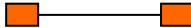  
*roX1Δ39* probe = 1.2 KB 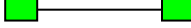  
 Internal Probe = 2.4 KB 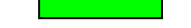

**B**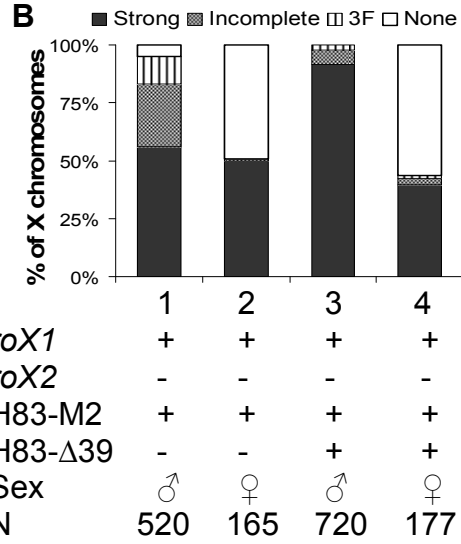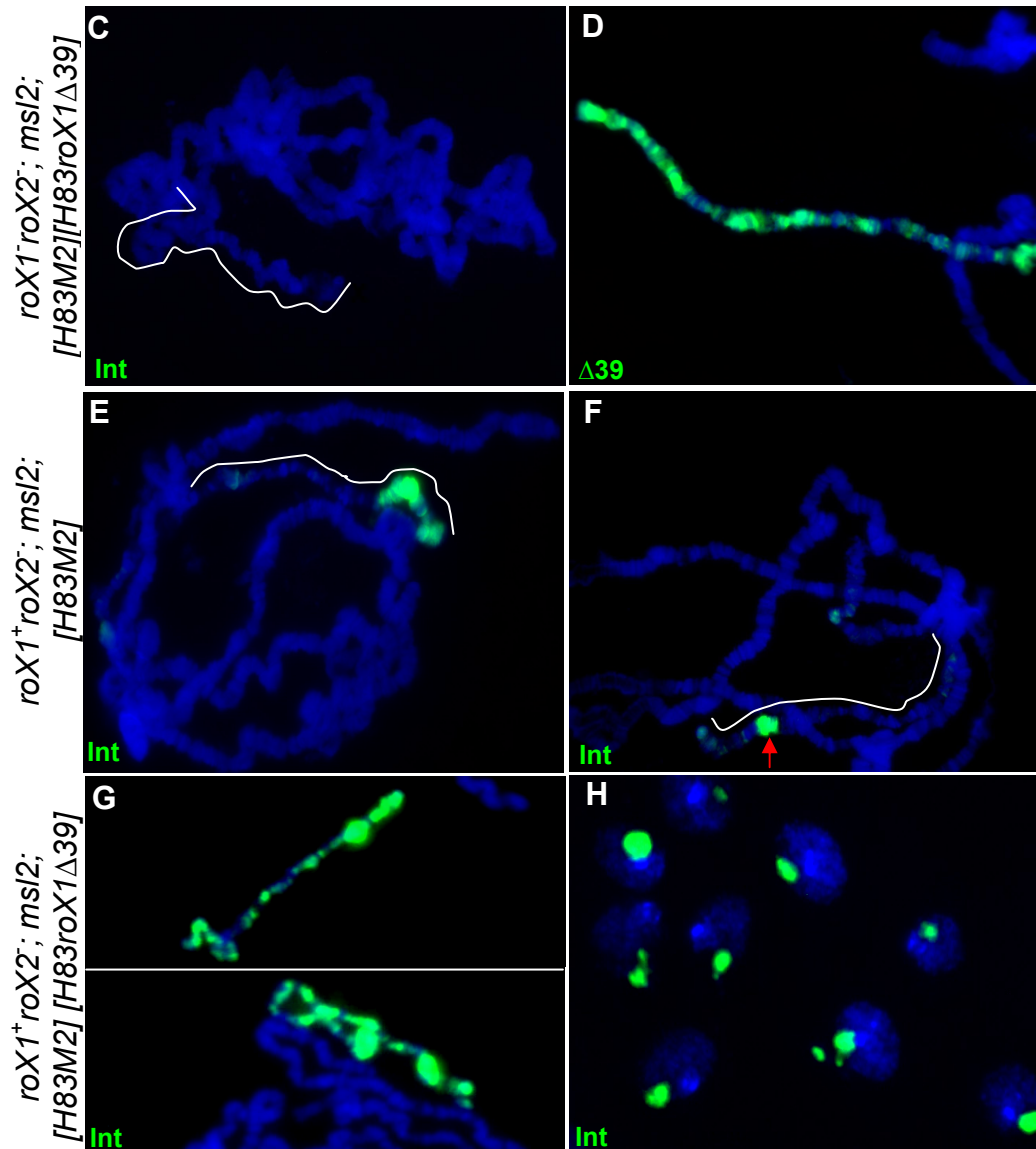

Supplement: Figure S1 — The [H83-roX1Δ39] transgene turns on endogenous roX1 in males. (A) roX1 transcripts (Orange) and antisense probes (green). (B) Quantification of roX1 hybridization over polytene X chromosomes: entire X (black), only distal X (gray, example E), a single band at the roX1 locus (hatched, example F red arrowhead), or no staining (white). N = nuclei counted. The [H83-Δ39roX1] transgene does not have an effect on females. (C) The internal probe does not hybridize to Δ39roX1 RNA, (D) but the roX1Δ39 probe does. White line delineates the X chromosome. (E) Males display a diverse pattern of roX painting, ranging from local spreading from the roX1 locus to (F) just a single band (indicated by the red arrow). (G) Δ39roX1 RNA (not visualized) helps [H83-M2] strongly activate endogenous roX1 (detected with internal probe) in almost all cells (two nuclei shown) (H) Similar roX1 staining was found in all squashed imaginal disc cells. (PDF) [file pgen.1002564.s001.pdf]

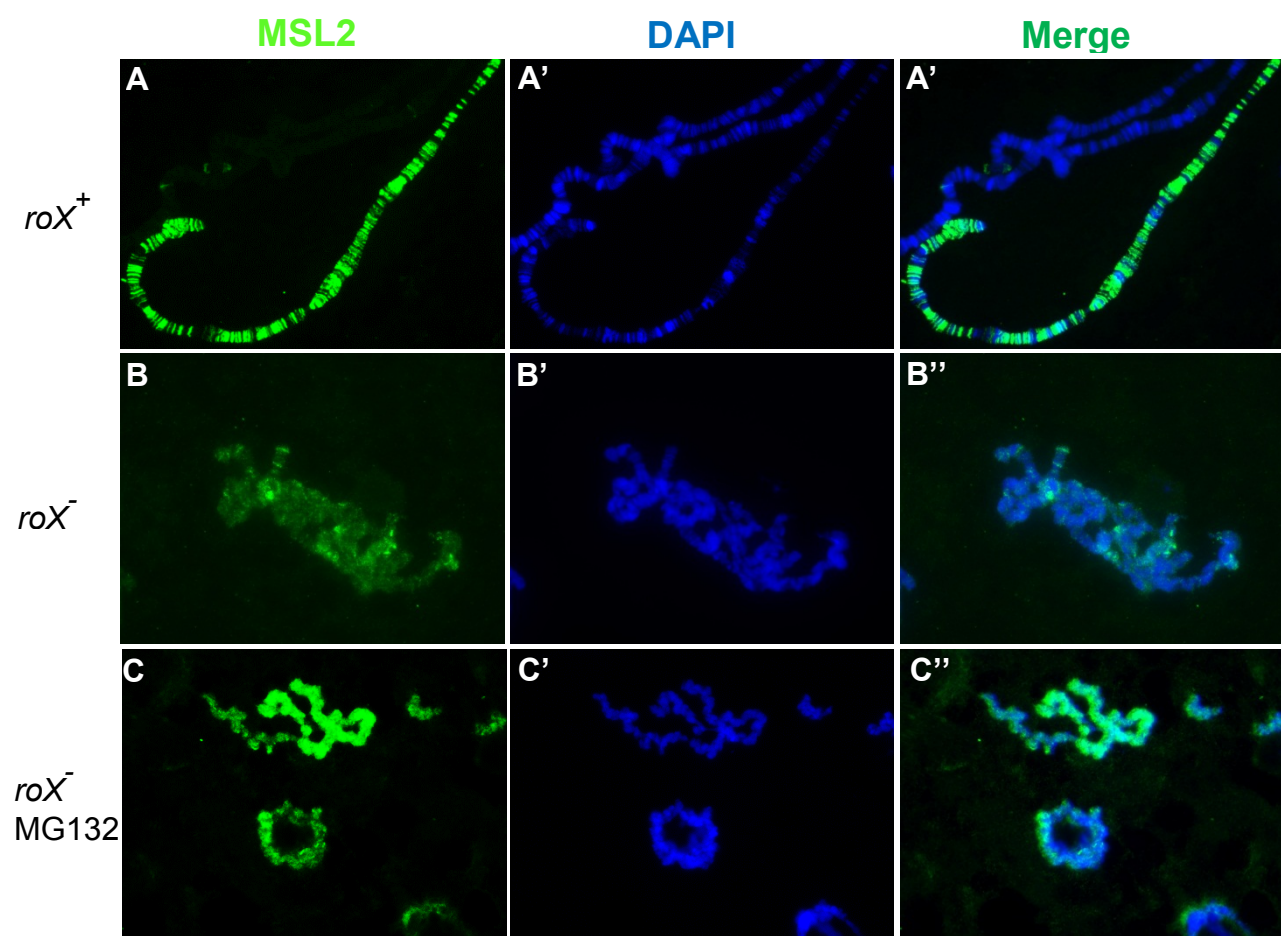

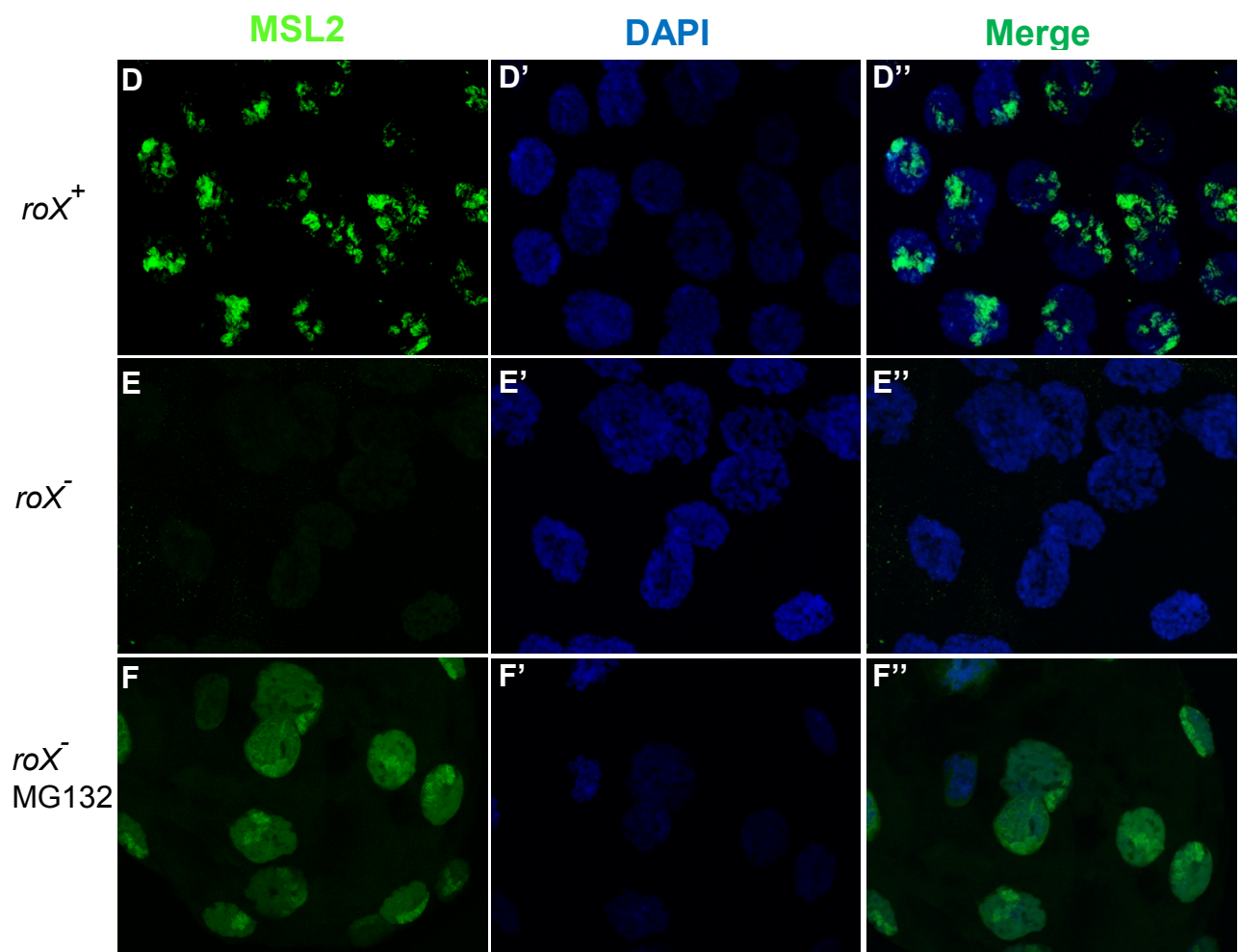

Supplement: Figure S2 — MSL immunostaining reveals that MSL2 binds indiscriminately to all the chromosomes in the absence of any roX RNA. (A) In the presence of roX RNA, MSL2 binds and paints the X chromosome only. (B) In the absence of roX RNA, the MSL proteins form incomplete complexes and binds to all the chromosomes, albeit poorly. (C) After treatment with MG132, a proteasome inhibitor, strong MSL2 binding can now be observed to occur throughout the nucleus. roX1− roX2− males are sick and do not survive till adulthood. However, sick and rare 3rd instar larvae can be obtained for salivary squashes although the chromosomes have extremely poor morphology and easily shattered during squashes. (D) The same experiment was repeated in whole mount salivary glands and MSL2 can be seen concentrated on the X chromosome. (E) At low resolution, MSL2 staining becomes undetectable in the absence of roX RNA (compared to B). (F) MSL2 can be seen binding to the entire nucleus when degradation is being inhibited. (PDF) [file pgen.1002564.s002.pdf]

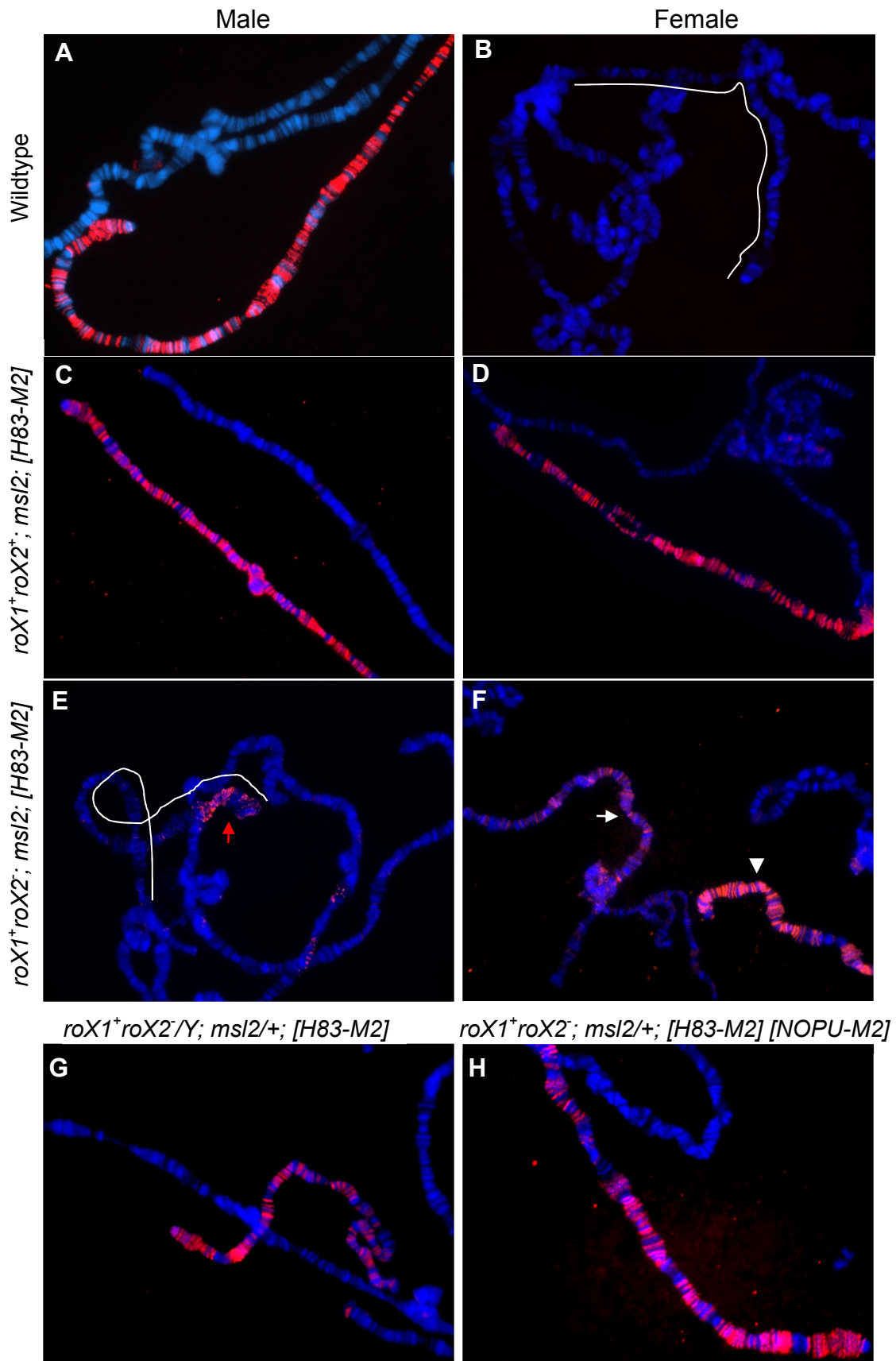

I

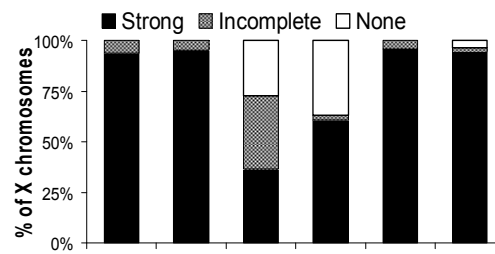

|                     | 1   | 2   | 3    | 4   | 5   | 6   |
|---------------------|-----|-----|------|-----|-----|-----|
| <i>roX1</i>         | +   | +   | +    | +   | +   | +   |
| <i>roX2</i>         | +   | +   | -    | -   | -   | -   |
| <i>msl2</i> locus   | -/- | -/- | -/-  | -/- | -/+ | -/+ |
| <i>H83-M2</i>       | +   | +   | +    | +   | +   | +   |
| <i>NOPU-M2</i>      | -   | -   | -    | -   | -   | +   |
| <i>H83-roX1cDNA</i> | -   | -   | -    | -   | -   | -   |
| Sex                 | ♂   | ♀   | ♂    | ♀   | ♂   | ♀   |
| N                   | 124 | 139 | 1475 | 732 | 142 | 163 |

Supplement: Figure S3 — MSL immunostaining of polytene chromosomes in [H83-M2] expressing larvae reveals mosaic establishment in DC. MSL immunostaining and DAPI is represented by red and blue respectively. (A) The MSL complex is bound along the male single X chromosome at hundreds of bands. (B) Due to the lack of MSL2, female do not have MSL binding to the X chromosome. (C–D) Painting of the X can be restored by the [H83-M2] transgene in msl2 mutant animals if both roX1 and roX2 are present. (E) The MSL complex fails to paint the entire X chromosome in males if roX2 is deleted. This cis-spreading phenomenon around the roX1 locus (indicated by red arrow) is similar to the autosomal spreading of roX1 transgene observed under low transcription rate (9). (F) In females, the X chromosome is either painted (arrowhead) or not painted (arrow). See Figure 1I for quantification. (G–H) Normal painting of the X is re-established when an endogenous copy of msl2 is restored. In females, this is achieved by co-expressing the [NOPU-M2] transgene. (I) The fraction of polytene nuclei displaying complete, partial, or no X MSL1 painting is shown. N = nuclei scored. Genotypes: (1–2) roX1+roX2+;msl2;[H83-M2]/+, (3–4) roX1+roX2−;msl2;[H83-M2]/+, (5) roX1+roX2−/Y;msl2/+;[H83-M2]/+, (6) roX1+roX2−;msl2/+;[NOPU-M2] [H83-M2]/+. (PDF) [file pgen.1002564.s003.pdf]

Male

Female

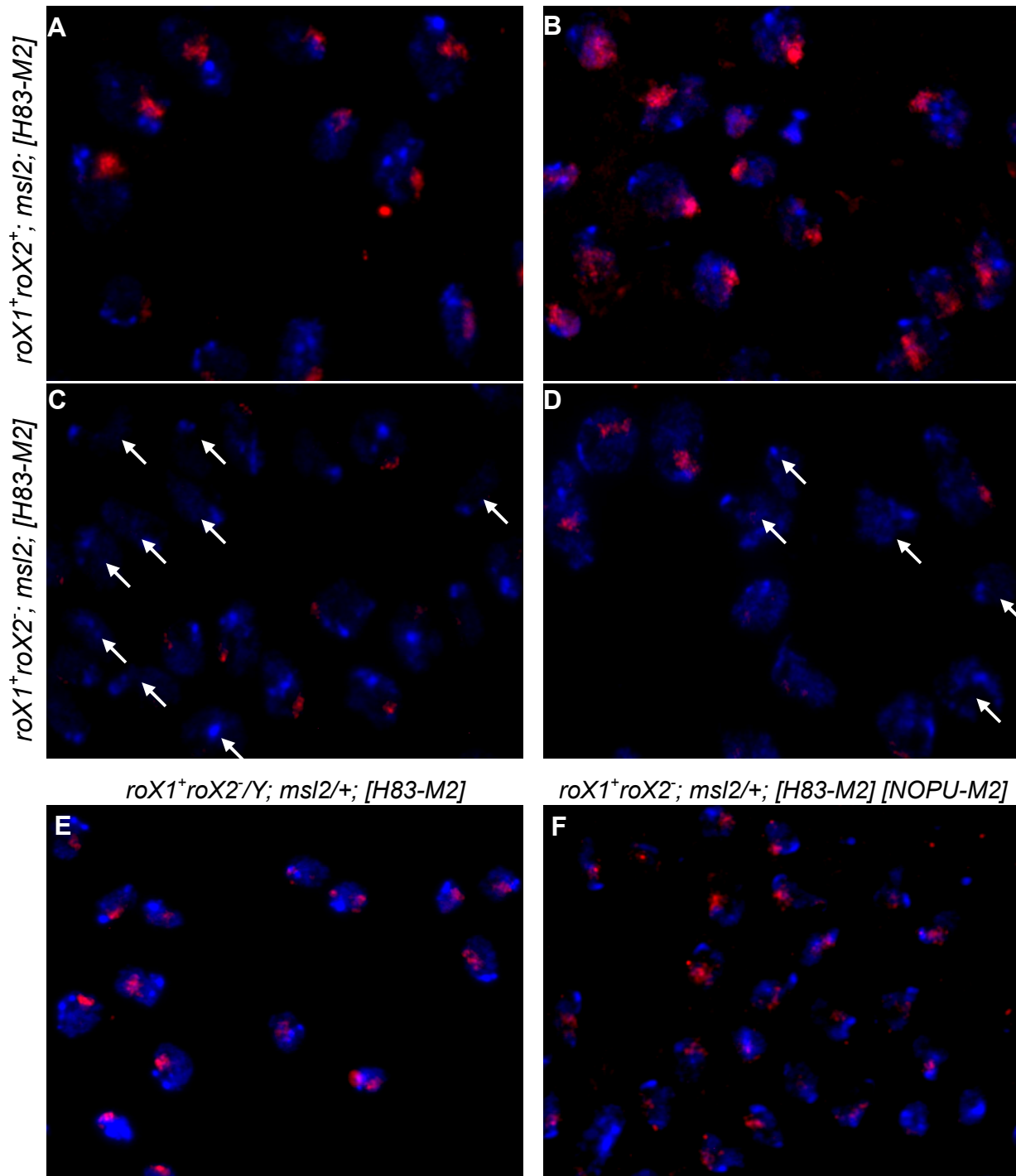

**G**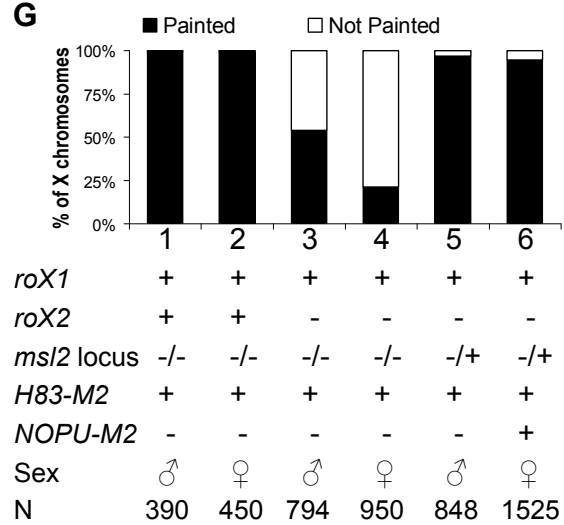

Supplement: Figure S4 — MSL2 immunostaining of imaginal disc cells in [H83-M2] expressing larvae reveals mosaic establishment of DC. (A–B) Painting in all nuclei is observed in animals relying upon the [H83-M2] transgene if both roX1 and roX2 are present. (C–D) Mosaic painting of the X chromosome is observed in both males and females when roX2 is deleted. White arrows indicated unpainted nucleus. (E–F) Normal painting of the X is re-established when an endogenous copy of msl2 is restored. In females, this is achieved by co-expressing the [NOPU-M2] transgene. (G) Cells with (black) and without (white) obvious subnuclear domain MSL2 staining taken from gently squashed discs were counted (Figure S1) N = cells counted. Genotypes: (1–2) roX1+roX2+;msl2;[H83-M2]/+, (3–4) roX1+roX2−;msl2;[H83-M2]/+, (5) roX1+roX2−/Y;msl2/+;[H83-M2]/+ male, (6) roX1+roX2−;msl2/+; [NOPU-M2][H83-M2]/+ female. (PDF) [file pgen.1002564.s004.pdf]

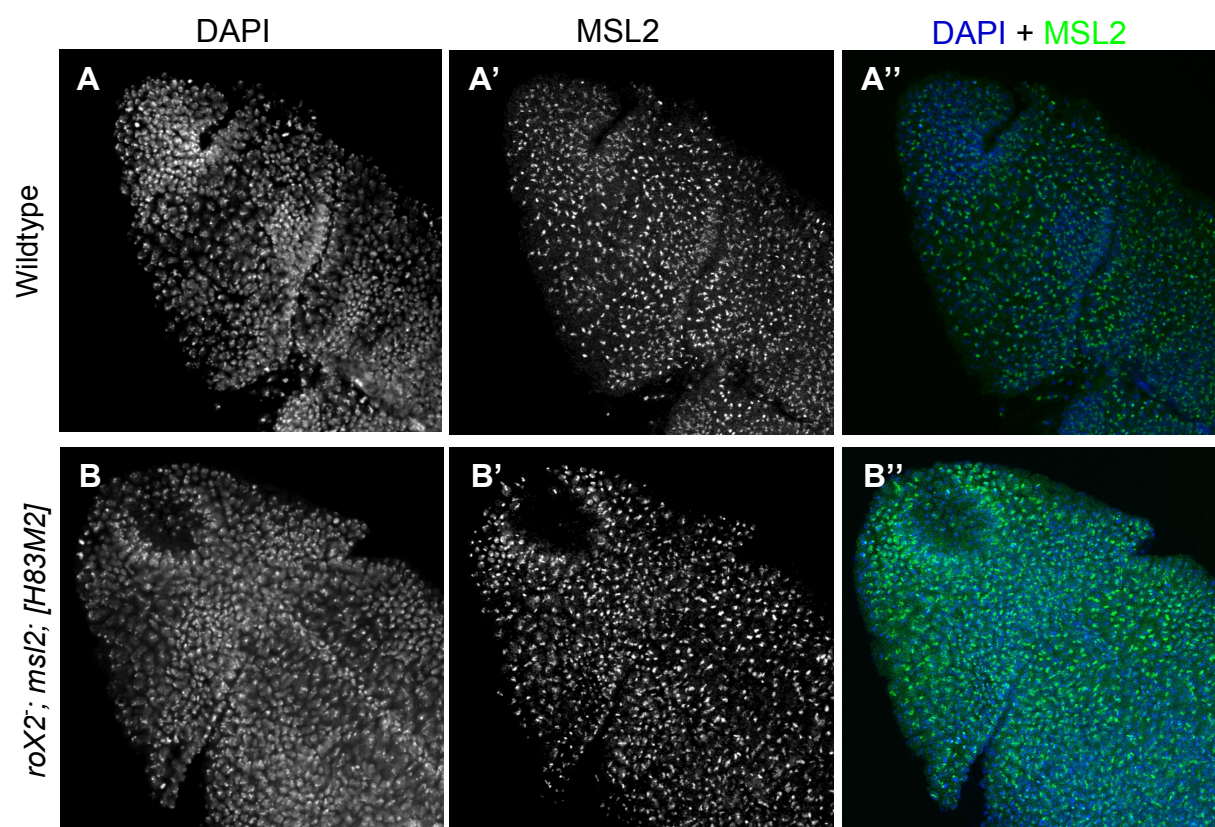

Supplement: Figure S5 — MSL2 immunostaining of embryos reveals that 100% of the cells expressed [H83-MSL2]. (A–A″) A wildtype embryo at the end of germband extension showing endogenous MSL2 expression detected in 100% of the cells (B–B″) A roX2−; msl2; [H83M2] embryos, expected to display a mosaic pattern of dosage compensation in imaginal discs and salivary glands by 3rd instar, shows no signs of mosaicism during early embryogenesis. (PDF) [file pgen.1002564.s005.pdf]

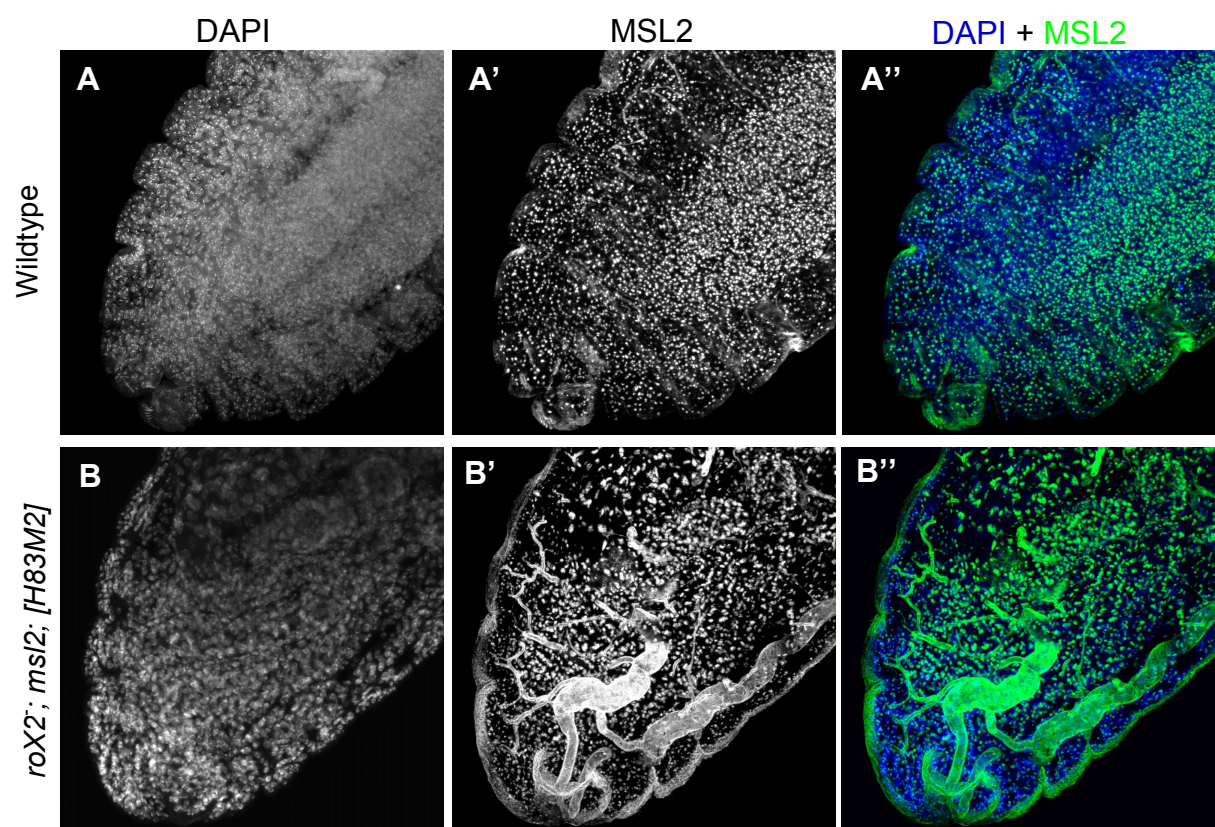

Supplement: Figure S6 — roX1 in situ hybridization of embryos reveals that 100% of the cells successfully establish dosage compensation. (A–A″) A wildtype embryo at about 20 hr AEL, has roX1 expression detected in 100% of the cells (B–B″) A similarly stage roX2−; msl2; [H83M2] embryos displaying no signs of mosaicism. (PDF) [file pgen.1002564.s006.pdf]

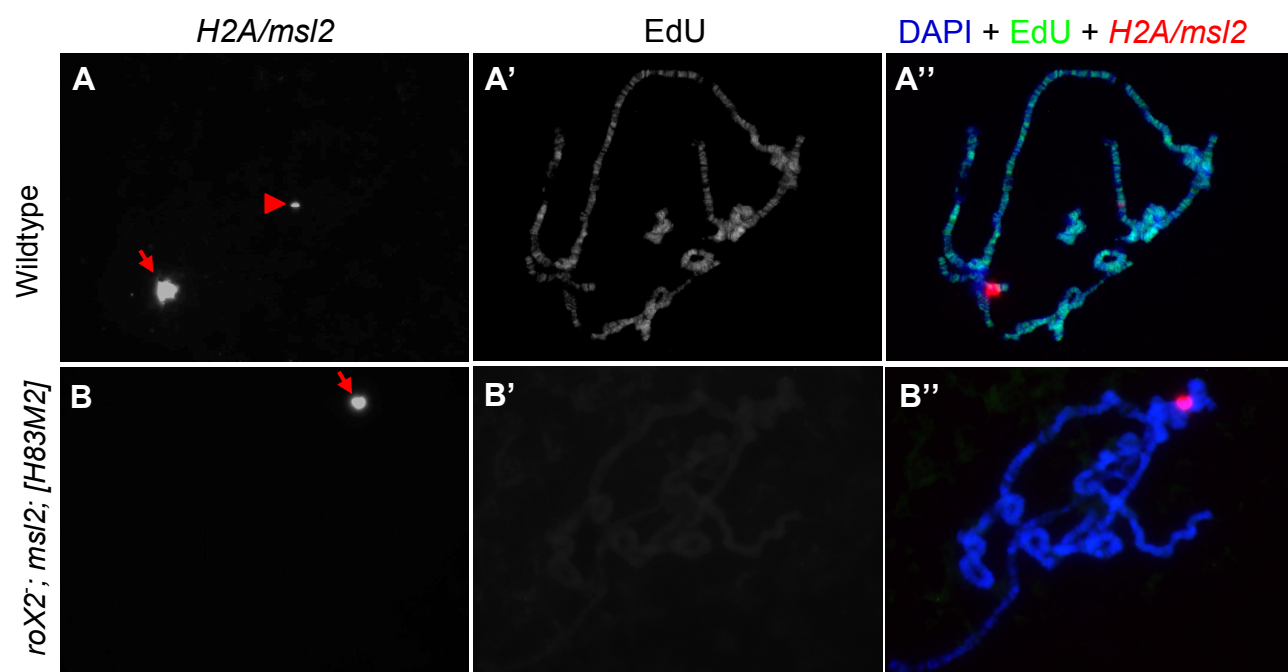

Supplement: Figure S7 — Histones transcripts not a good marker of S Phase. (A–A″) Nascent H2A (red arrow) and msl2 transcripts (red arrowhead) can be visualized at cytolocation 23F and 39 respectively via double ISH of polytene chromosomes with anti-sense msl2 and H2A RNA probes. (B) Although it is well documented that the transcription rate of histones decreases by 5 folds upon Hydroxurea treatment [38], it is hard to quantified using the TSA technique for ISH. (B′–B″) On the other hand, msl2 transcripts have completely disappeared upon HU treatment. (PDF) [file pgen.1002564.s007.pdf]
