# Supplementary material for: Autoregulation of the Drosophila Noncoding roX1 RNA Gene
Source: PLoS Genet. 2012 Mar 15;8(3):e1002564. doi: 10.1371/journal.pgen.1002564 (PMC3305356; doi:10.1371/journal.pgen.1002564)
Supplement: Figure S8 — Components of the DNA replication preinitiation complex bind transcriptional control regions within both roX1 and msl2 genes. (A) Both MCM and ORC2 bind the DHS control region within roX1 only in male cells. (B) Preinitiation complex is located near the promoters of many genes including msl2. Binding data shown for S2 cells. Screen captures of ChIP-seq data of the MacAlpine lab deposited in FlyBase Modencode. Y-axes not the same for different profiles. (PDF) [file pgen.1002564.s008.pdf]

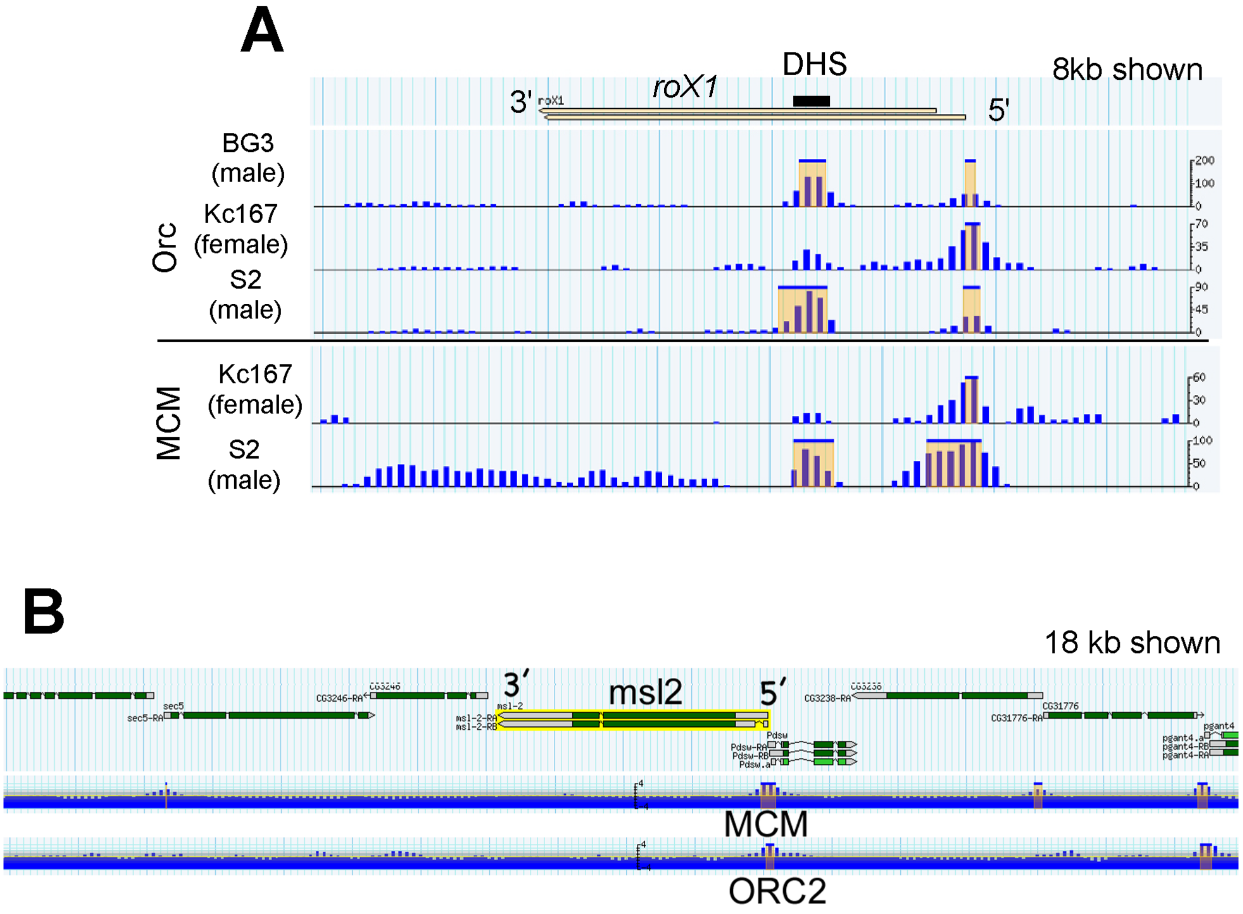
A
DHS
8kb shown
roX1
3'
5'
Orc
BG3
(male)
Kc167
(female)
S2
(male)
MCM
Kc167
(female)
S2
(male)
B
18 kb shown
3′
msl2
5′
MCM
ORC2
